# Semantic Linkages of Obsessions From an International Obsessive-Compulsive Disorder Mobile App Data Set: Big Data Analytics Study

**DOI:** 10.2196/25482

**Published:** 2021-06-21

**Authors:** Jamie D Feusner, Reza Mohideen, Stephen Smith, Ilyas Patanam, Anil Vaitla, Christopher Lam, Michelle Massi, Alex Leow

**Affiliations:** 1 Semel Institute for Neuroscience and Human Behavior University of California Los Angeles Los Angeles, CA United States; 2 Centre for Addiction and Mental Health Department of Psychiatry University of Toronto Toronto, ON Canada; 3 NOCD, LLC Chicago, IL United States; 4 Anxiety Therapy LA, Inc Los Angeles, CA United States; 5 Department of Psychiatry University of Illinois College of Medicine Chicago, IL United States

**Keywords:** OCD, natural language processing, clinical subtypes, semantic, word embedding, clustering

## Abstract

**Background:**

Obsessive-compulsive disorder (OCD) is characterized by recurrent intrusive thoughts, urges, or images (obsessions) and repetitive physical or mental behaviors (compulsions). Previous factor analytic and clustering studies suggest the presence of three or four subtypes of OCD symptoms. However, these studies have relied on predefined symptom checklists, which are limited in breadth and may be biased toward researchers’ previous conceptualizations of OCD.

**Objective:**

In this study, we examine a large data set of freely reported obsession symptoms obtained from an OCD mobile app as an alternative to uncovering potential OCD subtypes. From this, we examine data-driven clusters of obsessions based on their latent semantic relationships in the English language using word embeddings.

**Methods:**

We extracted free-text entry words describing obsessions in a large sample of users of a mobile app, *NOCD*. Semantic vector space modeling was applied using the Global Vectors for Word Representation algorithm. A domain-specific extension, *Mittens*, was also applied to enhance the corpus with OCD-specific words. The resulting representations provided linear substructures of the word vector in a 100-dimensional space. We applied principal component analysis to the 100-dimensional vector representation of the most frequent words, followed by k-means clustering to obtain clusters of related words.

**Results:**

We obtained 7001 unique words representing obsessions from 25,369 individuals. Heuristics for determining the optimal number of clusters pointed to a three-cluster solution for grouping subtypes of OCD. The first had themes relating to relationship and just-right; the second had themes relating to doubt and checking; and the third had themes relating to contamination, somatic, physical harm, and sexual harm. All three clusters showed close semantic relationships with each other in the central area of convergence, with themes relating to harm. An equal-sized split-sample analysis across individuals and a split-sample analysis over time both showed overall stable cluster solutions. Words in the third cluster were the most frequently occurring words, followed by words in the first cluster.

**Conclusions:**

The clustering of naturally acquired obsessional words resulted in three major groupings of semantic themes, which partially overlapped with predefined checklists from previous studies. Furthermore, the closeness of the overall embedded relationships across clusters and their central convergence on harm suggests that, at least at the level of self-reported obsessional thoughts, most obsessions have close semantic relationships. Harm to self or others may be an underlying organizing theme across many obsessions. Notably, *relationship*-themed words, not previously included in factor-analytic studies, clustered with *just-right* words. These novel insights have potential implications for understanding how an apparent multitude of obsessional symptoms are connected by underlying themes. This observation could aid exposure-based treatment approaches and could be used as a conceptual framework for future research.

## Introduction

### Background

Obsessive-compulsive disorder (OCD) is characterized by recurrent and persistent thoughts, urges, or images that are experienced as intrusive and inappropriate (obsessions) and that cause marked anxiety or distress and/or repetitive behaviors (compulsions) [1]. OCD has a lifetime prevalence of approximately 2%-2.3% worldwide [2,3] and is associated with functional impairment, poor quality of life, and increased use of health care services [4].

OCD symptoms can manifest in a variety of seemingly disparate ways [1]. Obsessions can be, for example, fears of contamination from one’s immediate environment, ego-dystonic thoughts that one might harm someone else in a violent or sexual manner despite not having a desire or intent to do so, believing that one has done something blasphemous, excessive doubts or concerns about one’s partner in a relationship, or having a difficult-to-describe need to feel that an object is placed *just right* in their environment. In response to these obsessions, patients might engage in different types of compulsive behaviors that could include, for example, handwashing, checking, praying, arranging items, mental self-reassurance, or avoiding situations that trigger obsessive thoughts.

This range of different symptoms has contributed to the notion that OCD may be a heterogeneous condition characterized by different *subtypes* [5,6]. If so, subtypes might have important implications for understanding the potentially different underlying neurobiology and may have clinical implications, such as different effective treatment approaches for different subtypes.

To date, multiple factor-analytic studies have been conducted on OCD to understand the aggregations of symptoms and establish different potential subtypes [7,8]. Most of these studies have focused on symptoms elicited from clinical interviews rather than underlying pathophysiological mechanisms or processes as the basis for subtyping [7]. Symptom category–based factor-analytic studies have demonstrated evidence for four dimensions or subgroups of obsessions: contamination, harming, symmetry or order, and hoarding (hoarding has since been reconceptualized in the Diagnostic and Statistical Manual-5 as a separate disorder) [9,10] or contamination or somatic, aggressive, sexual, or religious, symmetry or order, and hoarding [11,12]. Two studies found evidence for five latent structures: contamination, harming, symmetry or order, hoarding, and religious or sexual [13] or sexual or somatic [14]. Cluster analyses have also been applied to identify subgroups of OCD symptoms, with some finding similar results as the factor-analytic studies [15,16]; however, one analysis found evidence of additional obsessional symptom clusters of sexual or somatic and contamination or harming [17].

A meta-analysis of 21 studies and 5124 participants found four factors using category-level data: cleaning and contamination, aggressive, sexual, or somatic obsessions (*forbidden thoughts*), symmetry, and hoarding [8]. Using item-level data revealed a five-factor solution: symmetry, aggression or sexual or religious, contamination, aggression or checking, and somatic.

These studies and most approaches to assess OCD symptoms to date have relied on assessing the presence of symptoms by selection from the predefined sets of obsessions and compulsions, most commonly from the clinician-administered Yale-Brown Obsessive-Compulsive Scale Symptom Checklist (YBOCS-SC) [18]. The individual items on the YBOCS-SC and the clustering of these into 13 categories was originally established by clinical consensus. However, using a predefined list of symptoms may introduce bias as it could lead to patients and/or clinicians *fitting* the symptoms into the terms on the checklist, which are further arranged in predefined categories.

Apart from handwashing, the YBOCS-SC was shown in one study to have poor convergent validity with self-report measures [17]. However, this may be attributed to the incomplete representation of symptoms in the other self-report measures to which the YBOCS-SC was compared, which also has the limitation of having predefined checklists. For instance, relationship-related obsessions [19-24] pertaining to obsessions regarding the suitability of the relationship or the relationship partner are not in the YBOCS-SC. Another study found an incomplete correspondence between the YBOCS-SC and self-report measures [25]. Furthermore, its internal consistency reliability was low for symmetry or ordering and sexual or religious symptoms; however, it was adequate for contamination or handwashing and aggression or checking [26].

A potentially less biased approach to assess and classify the types of OCD symptoms could come from patients’ free responses rather than relying on checklists. However, using free responses to understand the relationships among these symptoms to determine if categories or factors exist poses a challenge because of the vast number of different possible responses. Very large samples of responses, from large numbers of individuals, are likely required to identify stable patterns because multiple repeated terms, or terms representing similar semantic themes of symptoms, would be needed to separate signals from noise.

Such large samples could be obtained using digitally obtained data, such as from mobile apps used by individuals with OCD [27]. Mobile digital sources of free-entry text could provide a valuable resource for uncovering naturalistic, unrestricted, spontaneous, and therefore relatively unbiased patterns of responses.

Although they are more difficult to classify as compared with checklists, the semantic relationships of freely entered words representing obsessions can be analyzed for their clustering relationships in the English language. A tool to perform this clustering is natural language processing (NLP)—a branch of artificial intelligence that attempts to bridge the gap between computers and humans using the natural language [28]. Some common use cases of NLP include language translation apps such as Google Translate and computerized personal assistants such as Siri and Google Assistant. Each of these apps rely on NLP to decipher and understand human languages to complete a task. Approaches using NLP could provide a better understanding of latent semantic themes that could form the organizing relationships of groupings of protean individual obsessional examples. As a result, we apply NLP to characterize obsessions in this first-of-its-kind study.

### Objectives

To explore this approach, we obtained free-entry data for obsessions from a mobile health treatment platform developed by NOCD [29]. The NOCD app, among other functions, provides users with a platform for setting up customizable exposure and response prevention (a form of cognitive behavioral therapy for OCD) exercises. The primary objective of this study is to determine the number, types, and relationships of semantically organized clusters of obsessions in a data-driven manner. To this end, we applied an NLP technique called word embedding to a large data set of words from a large sample of individuals using the NOCD app.

## Methods

**Figure 1:**
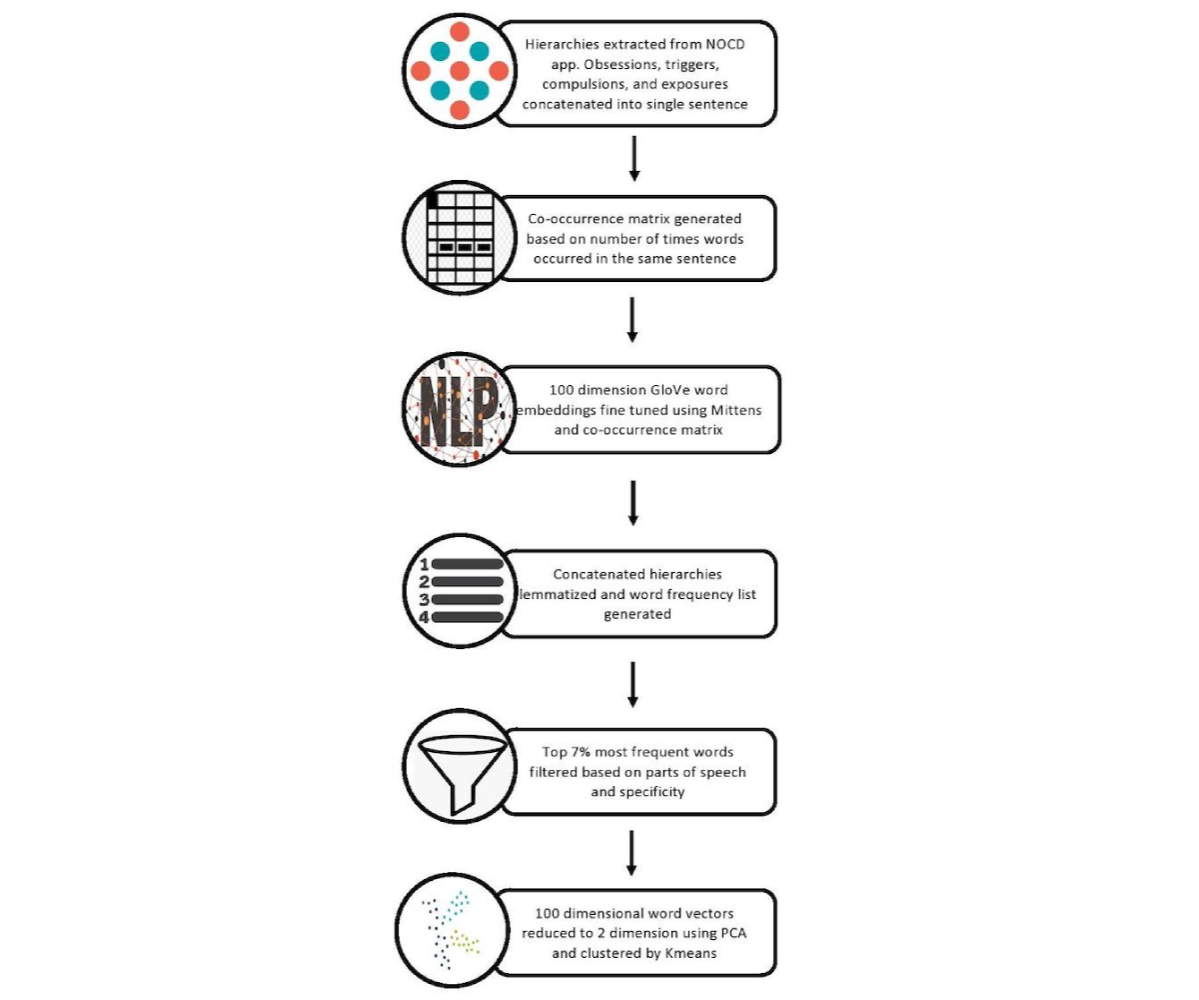
Data extraction and processing steps. GloVe: Global Vectors for Word Representation; PCA: principal component analysis.

An overview of the data extraction and following processing steps are shown in Figure 1.

### Data Extraction

We extracted data from freely entered words describing obsessions and their corresponding triggers, exposures, and compulsions by users of the iOS version of the mobile app NOCD who self-identified as having OCD (NOCD iOS app version 2.0.7-2.0.96). This app includes two features where users can input their current obsessions, namely, the “SOS” feature and the hierarchy, which can then be used for planned exposure exercises. Obsessions that had been inputted by users were assigned an anonymous user ID number, and all data were deidentified. NOCD users are required to be at least 17 years of age, but to preserve privacy, no specific demographic information was collected. Data were collected between March 22, 2018, and July 9, 2020.

### Data Preprocessing

Obsessions and descriptions of obsessions from their associated triggers, exposures, and compulsions were grouped together into a singular phrase. In addition, phrases were cleaned to remove punctuation, special characters, and spelling errors.

### Generating Word Embeddings—Global Vectors for Word Representation Algorithm and Mittens

A co-occurrence matrix of size N×N is generated by iterating over the aforementioned phrases and incrementing (*i*,*j*), where N is the number of unique words, and *i* and *j* correspond to two words that appear in the same phrase. Pretrained 100-dimension Global Vectors for Word Representation (GloVe) algorithm [30] word-embedding vectors were used in conjunction with the co-occurrence matrix as inputs to Mittens [31], which is an extension of GloVe for learning domain-specialized representations.

Thus, GloVe vectors, based on global word-to-word co-occurrence statistics from a 6 billion–word corpus, were fine-tuned based on the localized co-occurrence matrix. This provides a specialized representation of words and their relationships from the OCD-specific lexicon synthesized with pretrained representations from the general lexicon.

### Data Processing

To identify the root forms of words that may be inflections of each other, we performed lemmatization using Stanford CoreNLP—natural language software [32]. The entries were first parsed into single words. These words were assigned a frequency as to how often they occurred across all individuals’ entries and then sorted by the frequency of occurrence. The top percentage of most frequently occurring words was chosen; the top 7% was selected as it captured the most commonly occurring words balanced with the ability to visualize the cluster graphs.

These words were then filtered based on the parts of speech: adverbs, modals, third-person singular present verbs, gerunds, past participle verbs, *to*, prepositions, subordinating conjunctions, and personal pronouns. In addition, a clinician (JDF) reviewed the filtered list to further remove nonclinically relevant words, resulting in a total of 430 words.

### Clustering and Data Analysis

To visualize the 100-dimension latent semantic relationships of words in a 2D space, we applied principal component analysis to the 100-dimensional vector representation of the most frequent words and plotted the first two principal components. Furthermore, to identify clusters of related words in a data-driven manner, we performed k-means clustering. The optimal number of clusters was determined using the following heuristics: the silhouette coefficient [33], Elbow Method [34], Calinski-Harabasz Index [35], and the Davies-Bouldin index [36].

Once the optimal cluster number was determined, we compared the relative frequencies among the clusters of (1) unique obsessional words and (2) total obsessional words using the chi-square test.

## Results

### Characterization of the Data

We obtained 7001 unique words representing obsessions from 25,369 individuals aged 17 years and older, self-identified as having OCD across 108 countries. Most individuals were from the United States (18,315) with an additional 1335 North American entries from Canada and Mexico, 4134 from Europe, 557 from Asia, 100 from Africa, 137 from South America, and 791 from Australia and New Zealand.

In total, 94.99% (24,100/25,369) of users contributed no more than five obsessions each to the data set. Most users—16,988 (the mode)—contributed only one obsession; 4311 contributed two obsessions, 1861 contributed three obsessions, 854 contributed four obsessions, and 476 contributed five obsessions. There were two extreme outliers that contributed 120 and 174 obsessions. We removed these data before the analysis. In summary, although users could enter multiple words, given the large total number of obsessional words that were mapped and the fact that most users only entered one or two words, the overall results were unlikely to be biased by single users (Multimedia Appendix 1, Figure S5, shows a histogram of the number of word entries per user).

### Determining Cluster Size

The silhouette coefficient yielded optimal clusters with sizes of 3 and 5. The Elbow Method, Calinski-Harabasz index, and Davies-Bouldin index all pointed to k=3 as the optimal number of clusters (Multimedia Appendix 1, Figure S5).

### Relationships of Obsessional Words With Canonical OCD Symptom Factors

**Figure 2:**
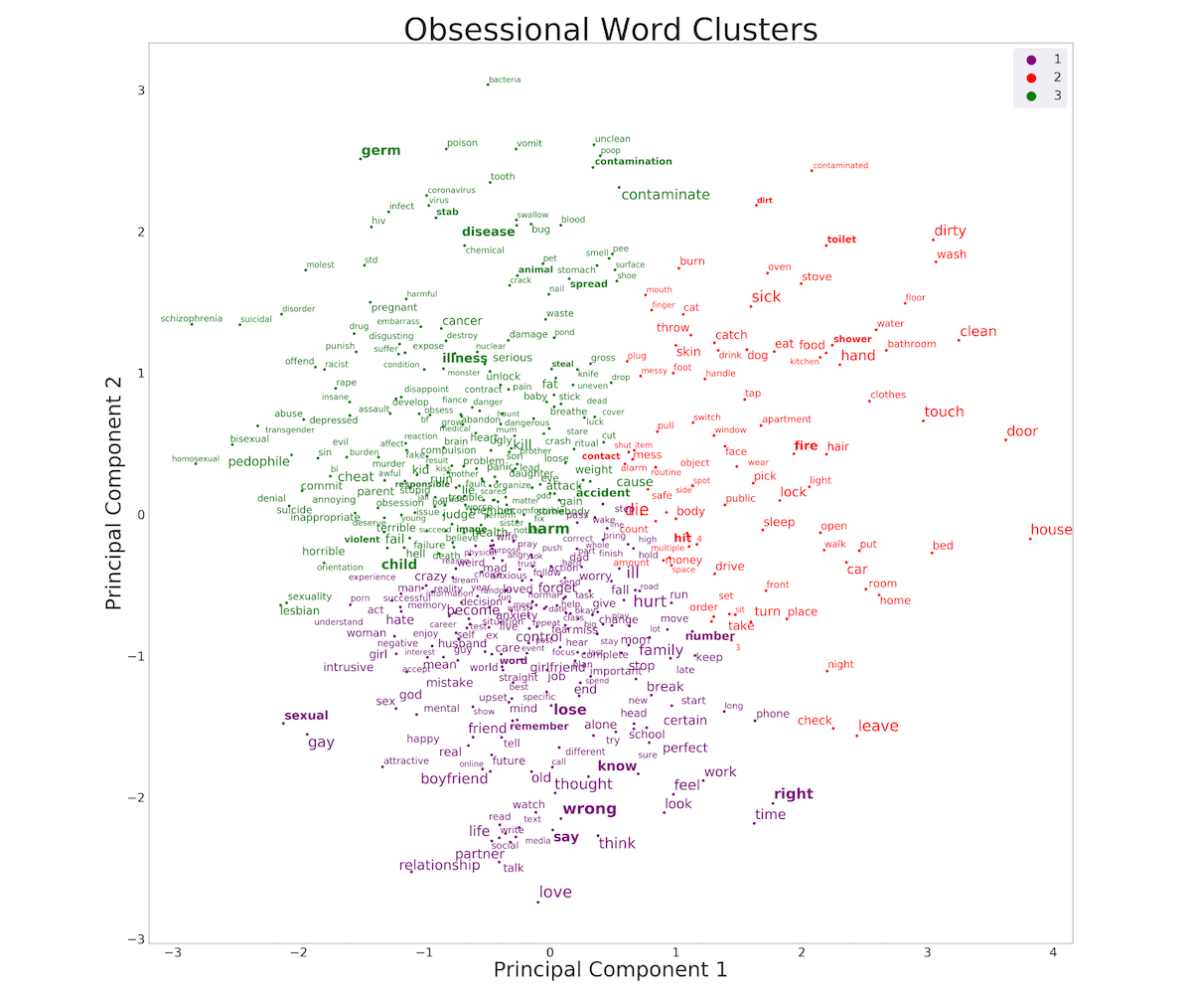
Frequently occurring obsessional words and their clustering, based on semantic relationships. The word embedding was trained on the entirety of the data set and clustered using k-means with k=3 clusters. The font is scaled according to the frequency of occurrence of each word. For reference, bolded words are those that also appear in the Yale-Brown Obsessive-Compulsive Scale Symptom Checklist.

**Figure 3:**
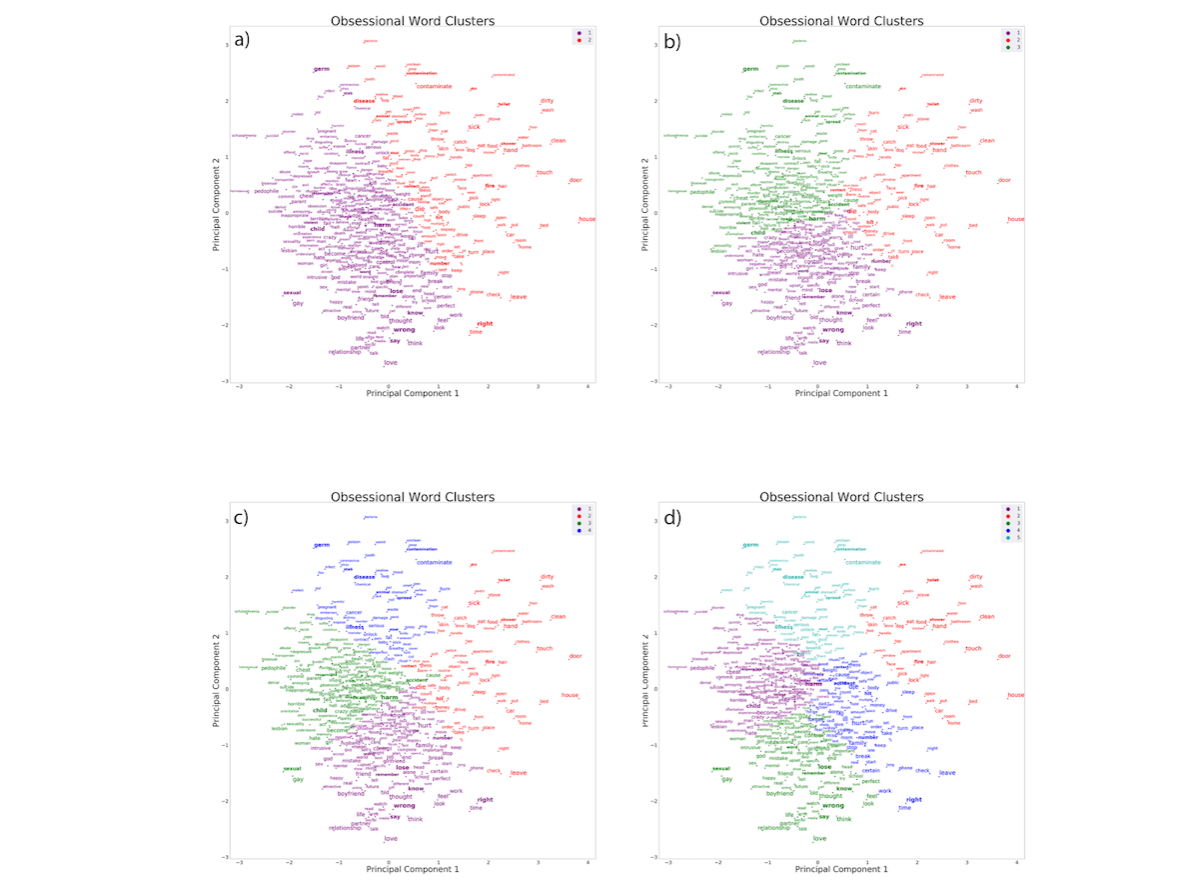
Frequently occurring obsessional words and their clustering, based on semantic relationships. The word embedding was trained on the entirety of the data set and clustered with k values of 2, 3, 4, and 5. The font is scaled according to the frequency of occurrence of each word. For reference, bolded words are those that also appear in the Yale-Brown Obsessive-Compulsive Scale Symptom Checklist. Observations of how the bolded words change clusters as k values change provide valuable insight into the similarities of OCD subtypes.

Next, we determined how the clusters of obsessions from our data-driven methods using freely reported obsessional words compared with previous factor-analytic and clustering studies that characterized symptom groupings based on rating scale checklists. To do this, we qualitatively examined which cluster appeared as OCD-specific words from the YBOCS-SC. We examined this for the optimal cluster solution of k=3 (Figure 2) [16] as well as for k=2, k=4, and k=5 clusters (Figure 3). Across all cluster solutions, there was a large, dense, central grouping with principal themes relating to harming of self or others (eg, *harm*, *accident*, and *hit*).

Furthermore, across k=3, k=4, and k=5 cluster solutions, the densest regions of each cluster were at a central convergence of the clusters, thereby suggesting closely grouped embedded relationships both within and across clusters (including on the borders between these clusters). Furthermore, cluster *3* in the k=3 solution that contained contamination and somatic-themed words (eg, *contamination*, *germ*, *disease*, and *illness*) and physical- and sexual-related harm words (eg, *harm*, *accident*, *child*, and *sexual*) split from each other in the k=4 solutions, suggesting that although these themes are related, distinctness is evident at the next level of separation.

### Split-Sample Repeat of the Clustering Analysis

We repeated the analysis in an equal-sized, nonchronological overlapping sample to control for any possible influences of minor changes in the NOCD app user interface that occurred during the period of data collection. The first sample was from March 22, 2018, to August 14, 2019 (11:18:04 PM), and the second sample was from August 14, 2019 (11:42:12 PM), to July 9, 2020, and each sample included 22,749 and 22,750 words, respectively.

The two aforementioned samples demonstrated similar clustering patterns, suggesting that the results were stable over time. To quantify this observation, we compared the 2D embeddings of the two groups by calculating the distance from a word to all other words on the graph. We performed this task for all words, creating a matrix of distances. Matrices for the two groups were compared using the Pearson correlation coefficient. Most correlations were above r=0.90, thereby demonstrating a high consistency of results across separate periods (Multimedia Appendix 1, Figure S14).

As the two abovementioned samples included some of the same individuals who entered obsessions in both the first and second periods, we additionally repeated the analysis in two nonoverlapping equal-sized user samples. The first sample contained 12,684 users with 22,510 obsessions and the second sample contained 12,685 users with 22,989 obsessions. By performing the same comparison of word-to-word matrices as for the chronological split sample, we found that the two split-by-users samples demonstrated very similar clustering patterns with the majority of correlations r≥0.90, suggesting that the results are reliable over subsets of users.

### Relative Frequency of Obsessional Words by Cluster

Comparing the three clusters on the total number of obsessional words, we observed 169 words in cluster 1 (just-right and relationship themes), 86 words in cluster 2 (doubt or checking themes), and 174 words in cluster 3 (contamination, somatic, or harm themes). All three clusters were significantly different from each other (χ^2^=34.2; *P*<.001). Furthermore, cluster 1 was significantly different from cluster 2 (χ^2^=27.4; *P*<.001) and cluster 3 (χ^2^=11.4; *P*<.001). Cluster 2 was significantly different from cluster 3 (χ^2^=29.4; *P*<.001). Thus, contamination, somatic, or harm words were more frequent than the just-right and relationship words and the doubt or checking words, and the just-right or relationship words were more frequent than the doubt or checking words.

## Discussion

### Principal Findings

In this large data set, we applied a data-driven approach and the NLP technique of word embedding to understand commonly occurring semantic themes of obsessions, freely reported by individuals using an OCD app. The optimal number of independent clusters that represents the relationships of the most frequently occurring obsessions was 3. Notably, the embedding patterns revealed that most obsessional words were closely grouped within and across clusters, including on the borders between clusters. Moreover, the densest region of each cluster is at a central convergence of the three, with principal themes relating to harm. This suggests that, at the level of self-reported obsessional thoughts, most obsessions have close semantic links with each other. Thus, although unique obsessions are protean, many examples, even across cluster *subtypes*, may actually have underlying latent relationships with each other.

### Observations of Semantic Themes of Clusters

To relate these findings to previous studies of OCD subtypes, we chose descriptive labels for the clusters from words from the widely used YBOCS-SC from six of the eight obsession categories: aggressive, sexual, religious, somatic, symmetry, and contamination (excluding hoarding and miscellaneous). Using these labels, the optimal cluster solution of three resulted in contamination-themed and physical- and sexual harm–themed obsessions occurring in the same cluster (cluster 3) and relationship and just-right themes occurring in the same cluster (cluster 1; Figure 2). Cluster 2 included many doubt-related obsessions typically associated with checking compulsions and a subset of contamination-related obsessions (eg, toilet, shower, sick, or dirty).

A notable overall observation is that the clustering algorithm produced topological patterns demonstrating a large but diffuse central cluster of harm-themed words, in addition to more diffuse and distanced words that represent contamination themes and doubt-related obsessions typically associated with checking compulsions (eg, fire, house, door, car, or lock). This is most readily apparent at the lowest level of clustering (k=2; Figure 3). As the number of clusters increases from k=3 to k=4 to k=5, some contamination-themed words joined with somatic-related themes (cluster 3 for k=3; Figure 3), which subsequently split into two different clusters (clusters 3 and 4 for k=4; Figure 3). At k=5, the original harm cluster from k=2 split into three clusters (clusters 1, 3, and 4), comprising words with themes related to sexual, sexual orientation, and relationships (cluster 1); *just-right* (cluster 3); and physical harm and a subset of doubt or checking (cluster 4).

This latter observation that a subset of doubt or checking words split off into two separate clusters at this level is consistent with a previous factor-analytic study finding that *aggressive* obsessions and checking compulsions tend to show instability on which factors they load on [37]. This progressive splitting of groups of obsessions as the cluster number increases provides unique insights into how closely some obsessional themes are related to each other based on their semantic relationships embedded in a 100-dimensional space.

These results are largely consistent with the findings of previous factor-analytic studies. A previous meta-analysis [8] of factor-level results similarly suggested a three-factor solution (not including hoarding symptoms); however, the item-level results pointed to five factors. There are several possible reasons why results differ, at least partially, from previous studies. First, this study represents an obsession from a much larger sample size: 25,369 individuals. The previous meta-analysis included a total of 5124 individuals; yet, individual study sizes ranged from 45 to 615. Thus, this study might have sampled a broader population.

Furthermore, as described in the Introduction section, previous factor-analytic and clustering studies used data collected with existing scales that include predefined checklists and categories, often from the YBOCS-SC. This could have the effect of creating a conceptual framework into which patients (and clinicians and researchers) may *shoe-horn* their experiences and symptoms. In addition, if a patient has an obsessional preoccupation that is not listed on the scale, then the patient, clinician, or researcher may not identify it as an OCD symptom. Freely entered text, as the NOCD app allows, mitigates this limitation. Notwithstanding, many individuals using the app may have already been exposed to commonly used lexicons regarding OCD subtypes, from clinicians, educational material, the media, or even the NOCD app itself because the online *community* forum mentions OCD subtypes.

Additional factors might account for why results partially diverge from previous factor-analytic and clustering studies. Some patients may feel embarrassed, ashamed, or even fearful of the consequences of divulging certain *taboo* obsessional thoughts, such as blasphemous, pedophilic, other sexual, or violent themes in front of a clinician or researcher. Such individuals may find it easier to enter these, on their own, in an app. In addition, in the online community forum in NOCD, people can find others who share similar thoughts. Once they have identified that what they experience is likely an OCD symptom, because it is shared by others, this could facilitate them entering the obsessional words related to these themes in the *hierarchy* part of the app. In sum, this approach of mapping the embedded latent relationships of obsessions partially recapitulates factor-analytic studies’ findings; yet, the differences might reflect a more unconstrained capturing of naturally occurring obsessions.

The split-sample test over time and across unique individuals demonstrated overall stable results. This stability across time suggests that the semantic themes of obsessional words entered by users are mostly invariant to periodic updates and fixes that happened with NOCD, as is standard with the most widely used mobile apps. In addition, early adopters of apps in general may have certain characteristics that differ from later adopters [38]. However, the stability across the split sample demonstrates that the results hold across these potentially different sets of individuals.

### Relative Frequencies of Obsessions Across Clusters

Earlier studies that categorized patients with OCD according to primary compulsions observed that contamination obsessions were the most common [39]. A subsequent study found that cleaning compulsions (therefore most often associated with contamination fears) were almost twice as common as checking compulsions (most often associated with harm or doubt obsessions) and more than four times as common as obsessions without physical compulsions in people with OCD seeking treatment [40]. A community assessment of OCD from the National Comorbidity Survey Replication included 2073 people and administered the YBOCS [2]. The results suggested that obsessions leading to checking (typically caused by obsessional fear of harm or doubt) are more common than contamination fears. The latter finding could have included the types of harm obsessions that were found to be highly represented in this study’s data set (clusters 2 and 3 in Figure 3), although it is unclear from the data collected in that study.

In this study’s sample, obsessions with harm themes were represented in a much higher proportion than in previous studies. Although harm-, contamination-, and somatic-themed words were clustered together in the k=3 grouping, the harm and contamination or somatic themes split and harm themes were nearly twice as common (n=138) as contamination or somatic words (n=70) at k=4. As most individuals in the current sample contributed only one obsession, this might represent their primary symptom, although this was not specifically ascertained. One interpretation of this finding is that this could represent a truer reflection of the relative proportions of these obsessional themes in the larger population of those with OCD, particularly because this study had a sample size that was an order of magnitude larger than the National Comorbidity Survey Replication. Furthermore, previous studies may not have found such relative proportions because of the limited thematic content of the YBOCS-SC or other instruments used to collect these data (as mentioned earlier). In addition, as mentioned earlier, *taboo* themes often relate to harming others and might appear more frequently in this data set obtained via relatively anonymous entry as compared with other data sets from population-based studies that involved researchers directly querying participants about subtypes.

An alternative interpretation is that there may be an overrepresentation of individuals who use the NOCD app who have harm obsessions. If this is the case, then it could be related to the stigmatization and *taboo* nature of these themes. Individuals with unwanted obsessional thoughts that are taboo in most societies and cultures, such as sexual thoughts about children, incest, physically harming someone, or blasphemous religious thoughts, may not readily share them with clinicians or researchers. However, they may feel more willing to share these in an app, which is a more anonymous experience. In addition, the NOCD app includes an online community forum in which other individuals share topics such as obsessional thoughts. This may help individuals not only realize that these thoughts could be related to OCD (they may not have found exact examples of their particular obsession elsewhere) but also the stigmatization of sharing them and entering them in the app could be reduced.

Another possibility that could lead to the overrepresentation of individuals with *harm* themes among NOCD users is that they might not have received effective treatment elsewhere and therefore gravitated toward NOCD as an alternative to try to find help. A study by primary care physicians demonstrated that OCD was misdiagnosed as either another psychiatric or psychological condition or no diagnosis 50.5% of the time [41]. Furthermore, in that study, obsessions related to homosexuality, aggression, and pedophilia were misdiagnosed ≥70% of the time. A similar study by clinical psychologists found that OCD was misdiagnosed 38.9% of the time and obsessions about the taboo thoughts of homosexuality, sexual obsessions about children, aggressive obsessions, and religious obsessions were more frequently misdiagnosed than contamination obsessions [42]. Even if diagnosed correctly, clinicians might find these types of symptoms difficult to understand and/or difficult to treat with exposure and response prevention because the themes are outside of the well-known *textbook* examples of contamination fears and checking compulsions, many of which have mental rather than physical compulsions or primarily engage in avoidance behaviors.

### Relationship-Themed Obsessions

Obsessions related to relationships have recently received attention in clinical and research settings [19-24], spawning the term “relationship OCD.” Although the *Diagnostic and Statistical Manual of Mental Disorders, Fifth Edition* [1] does not explicitly describe “relationship obsessions,” it provides an example under Other Specified Obsessive-Compulsive and Related Disorder, “Obsessional jealousy...a nondelusional preoccupation with a partner’s perceived infidelity.” Other partner-centered obsessional themes include obsessions about a partner’s flaws, such as intelligence, social aptitude, and morality [21]. Additional themes are centered on the relationship itself and can include obsessional doubting about whether one’s relationship is a *good* or *ideal* relationship. In addition to having obsessional *thoughts* about relationships, this can also include intrusive images, urges, or *not right* feelings.

To the best of our knowledge, this study is the first to characterize relationship-themed obsessions using clustering or factor-analytic approaches. Many such relationship themes appear in the same cluster as “just-right” obsessions, sometimes referred to as “not-just-right-experiences” [43]. This suggests the possibility that a driving force for many with relationship-themed obsessions could be that something about it does not *feel right*, rather than, for example, other catastrophic or otherwise negative consequences related to the relationship itself or one’s partner.

### Potential Clinical Implications

There are several potentially important insights that this study provides to the semantic relationships of obsessional thoughts. These have potential clinical assessment and treatment implications for understanding how an apparent multitude of *surface* obsessional symptoms are connected by underlying themes. This could assist clinicians during the assessment phase to facilitate a more focused and personalized inquiry about the presence of additional obsessions that may be semantically related to those reported. This could be helpful, particularly because patients with OCD are sometimes unaware that a particular thought is an obsession. Otherwise, it is difficult and time consuming for a clinician to guess what other obsessions they might have or to present patients with an extremely long and unfocused list of obsessions to choose from.

The findings from this study could also aid in planning exposure-based treatment approaches, for example, therapists could potentially map their patient’s primary obsessional themes to understand what are nearest-neighbor themes that might tap into a yet-unexplored core obsessional fear. A specific example of the potential utility of these findings could be to help clinicians explore whether the underlying feeling or emotion associated with a relationship-themed obsession is a not-just-right experiences versus the fear of a negative consequence of the relationship.

### Limitations, Strengths, and Future Directions

One of the limitations of this study is that although the data come from individuals who sought out and used therapeutic tools on an OCD app, we cannot confirm whether they met the diagnostic criteria for OCD because they did not undergo a diagnostic evaluation. It would be useful to repeat the procedures and analysis in a clinically diagnosed OCD sample; however, achieving a similar sample size would be extremely challenging. Therefore, this study’s results apply to those using the NOCD app, implying that they self-identify as having OCD or an OCD-like problem and could include those with other obsessive-compulsive and related disorders, for example, body dysmorphic disorder or other disorders with prominent obsessional thinking (eg, anorexia nervosa or illness anxiety disorder). This approach is in line with alternative transdiagnostic or dimensional strategies to study psychiatric illnesses, such as the National Institute for Mental Health’s Research Domain Criteria [44] framework. Another limitation is that we only examined postings in English. Although the majority of postings were in the English language, there were postings in other languages as well because NOCD is available and used worldwide. Another limitation is that we had limited demographic information because the data were strictly deidentified, limiting our ability to characterize demographic subgroups of individuals with OCD or determine whether these data represented the same gender distributions that are observed in the population of those with OCD. A further limitation is that the sample was limited to those aged 17 years and older. Whether the results can be generalized to children with OCD is unknown.

Finally, because of the variable nature of OCD and variability in the number of obsessions a user will choose to enter in the NOCD app, there was a skewed distribution of the frequency of obsessions across users (Multimedia Appendix 1, Figure S1). Thus, a small number of users theoretically could have influenced the results both at the stages of Mittens enhancement of GloVe embedding and of the comparison of frequencies of obsessions among clusters. However, to mitigate this, we removed the two extreme outliers. Furthermore, the split-sample results across equal numbers of unique users showed stable clusters and relative frequencies of obsessions, so it is unlikely that the main outcomes were influenced by a small number of individual users.

There are several important directions for future research. To understand the dynamics of semantic themes—specifically, if and how they change over time within individuals—it would be useful to obtain longitudinal data. This would allow, for example, an exploration of whether obsessional symptoms are stable in individuals or stable within semantic clusters or whether they cross over into different semantic themes. In addition, semantically defined OCD subtypes could be used as a conceptual framework to explore whether there are corresponding differences in the underlying neurobiology.

### Conclusions

In this study, we analyzed the semantic relationships of obsessional words freely entered in a mobile OCD app using a large data set. This novel, data-driven method provides unique insights into the relationships of obsessional themes and identifies potential OCD subtypes. This method is distinct from traditional characterizations of phenomenology in OCD; it is not easily achieved in clinical settings or in-person research settings and circumvents the limitations and biases of pre-existing clinical and research conceptualizations of established OCD subtypes.

## Appendix

Multimedia Appendix 1 Supplemental document that contains visualizations of analysis pertaining to characterization of the data in terms of demographics and sample size, determination of cluster size, and split-sample repeat of clustering analysis.

## Abbreviations

GloVe: Global Vectors for Word Representation
NLP: natural language processing
OCD: obsessive-compulsive disorder
YBOCS-SC: Yale-Brown Obsessive-Compulsive Scale Symptom Checklist
