# Supplementary material for: Semantic Linkages of Obsessions From an International Obsessive-Compulsive Disorder Mobile App Data Set: Big Data Analytics Study
Source: J Med Internet Res. 2021 Jun 21;23(6):e25482. doi: 10.2196/25482 (PMC8277342; doi:10.2196/25482)
Supplement: Multimedia Appendix 1 [file jmir_v23i6e25482_app1.pdf]

Supplemental Materials for

Semantic Relationships of Obsessions: Clustering and Frequencies of Obsessional Symptoms from a Large International Obsessive-Compulsive Disorder Mobile Application Dataset

Jamie D. Feusner[1*], Reza Mohideen[2*], Stephen Smith[2], Ilyas Patanam[2], Anil Vaitla[2], Christopher Lam[2], Michelle Massi[3], Alex Leow[4]

Author Affiliations:
[1]Department of Psychiatry and Biobehavioral Sciences, University of California Los Angeles, CA, USA
[2]NOCD, LLC, Chicago, IL, 60611, USA
[3]Anxiety Therapy L.A., Los Angeles, CA 90025, USA
[4]Department of Psychiatry, University of Illinois College of Medicine, Chicago, IL, 60612, USA
*These authors contributed equally

Table of Contents

Figure S1………… Page 2
Figure S2………… Page 3
Figure S3………… Page 4
Figure S4………… Page 5
Figure S5………… Page 6
Figure S6………… Page 7
Figure S7………… Page 8
Figure S8………… Page 9
Figure S9………… Page 10
Figure S10………… Page 11
Figure S11………… Page 12
Figure S12………… Page 13
Figure S13………… Page 14
Figure S14………… Page 15
Figure S15a………… Page 16
Figure S15b………… Page 16
Figure S16………… Page 18
Figure S17………… Page 19
Figure S18………… Page 20
Figure S19………… Page 21
Figure S20………… Page 22
Figure S21………… Page 23
Figure S22………… Page 24
Figure S23………… Page 25
Figure S24………… Page 26

Figure S25a... Page 27
Figure S25b... Page 27
Figure S26.. Page 27

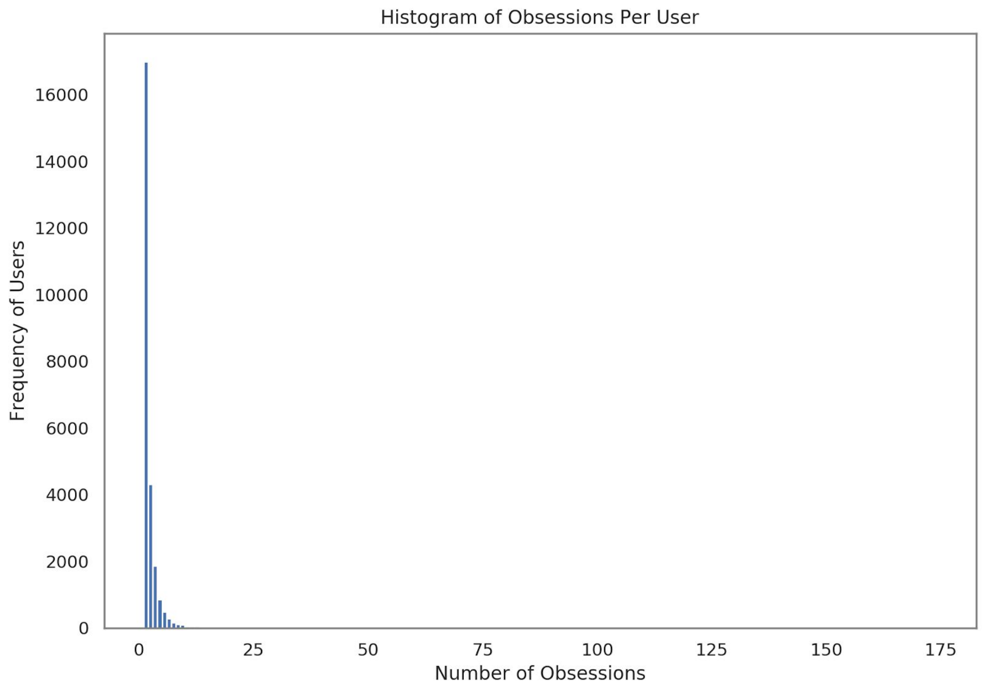

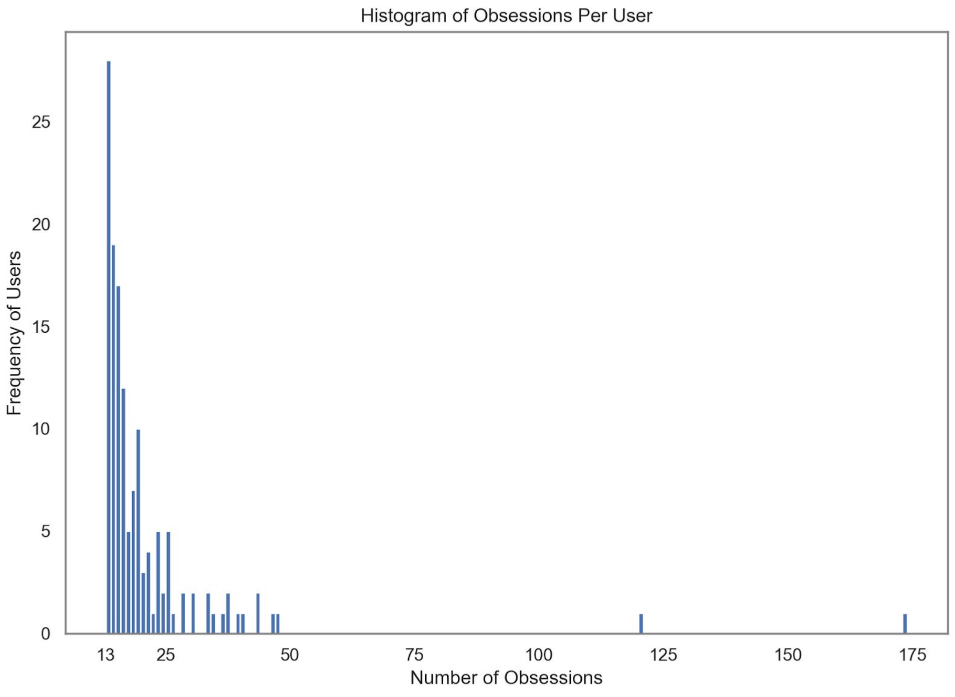

Figure S1. Histogram of frequency of users with the number of obsessions that they inputted. Top histogram shows an overview of all obsessions that were inputted from 0 to 175. Bottom histogram represents a magnified view of obsessions from 13 to 175, to better show the tail of the distribution and outliers.

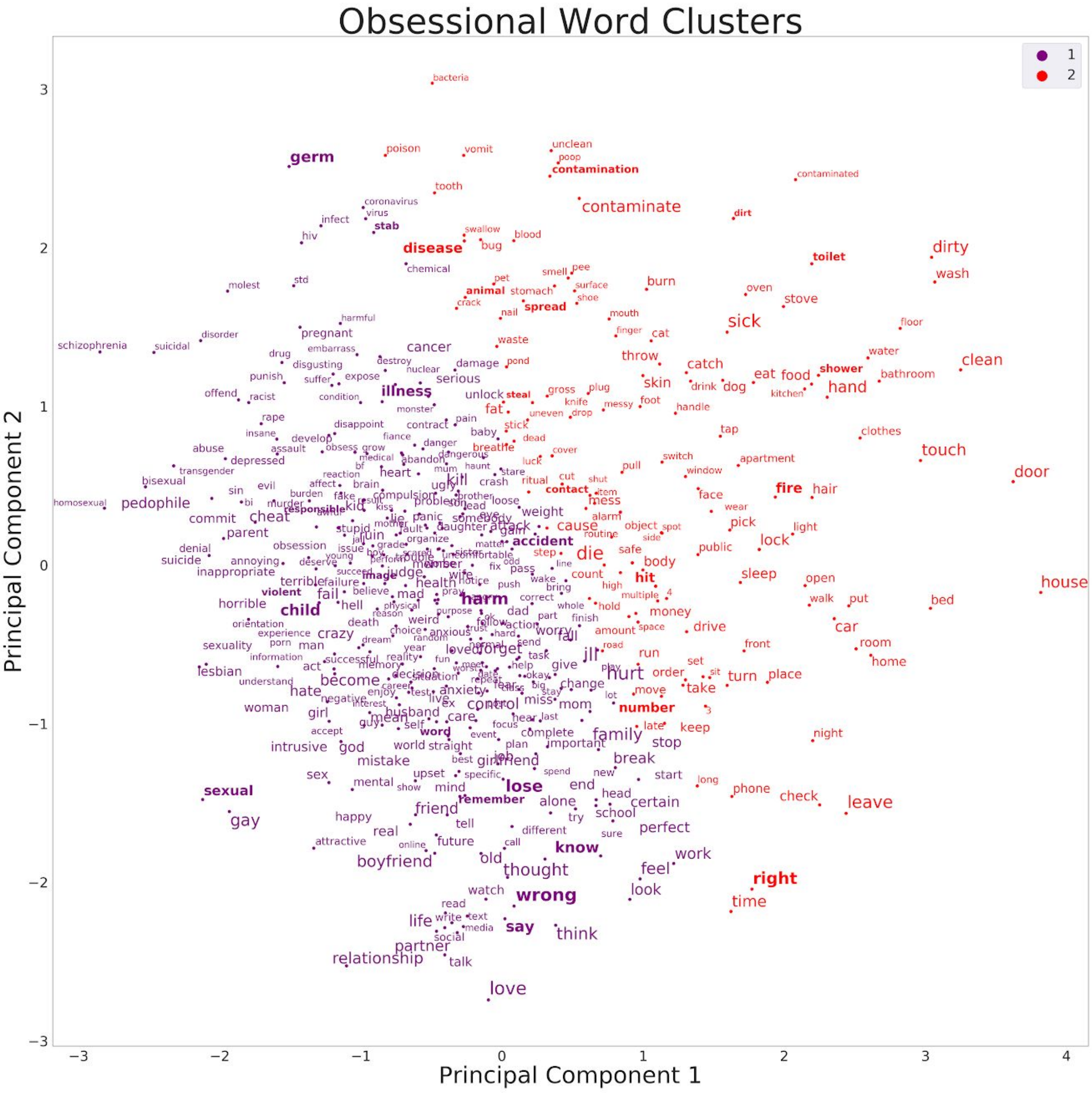

Figure S2. Top 7% most frequently inputted obsessional words. The embedding was trained on the entirety of the dataset and clustered using k-means with k = 2 clusters. (Note: this is simply a larger version of Fig. 3a in the main text.) For the equivalent depiction of results for k = 3, please see Fig. 2 in the main text. The font is scaled according to the frequency of occurrence of each word. For reference, **bolded** words are those that also appear in the Yale-Brown Obsessive-Compulsive Scale Symptom Checklist (YBOCS-SC) (Goodman et al. 1989).

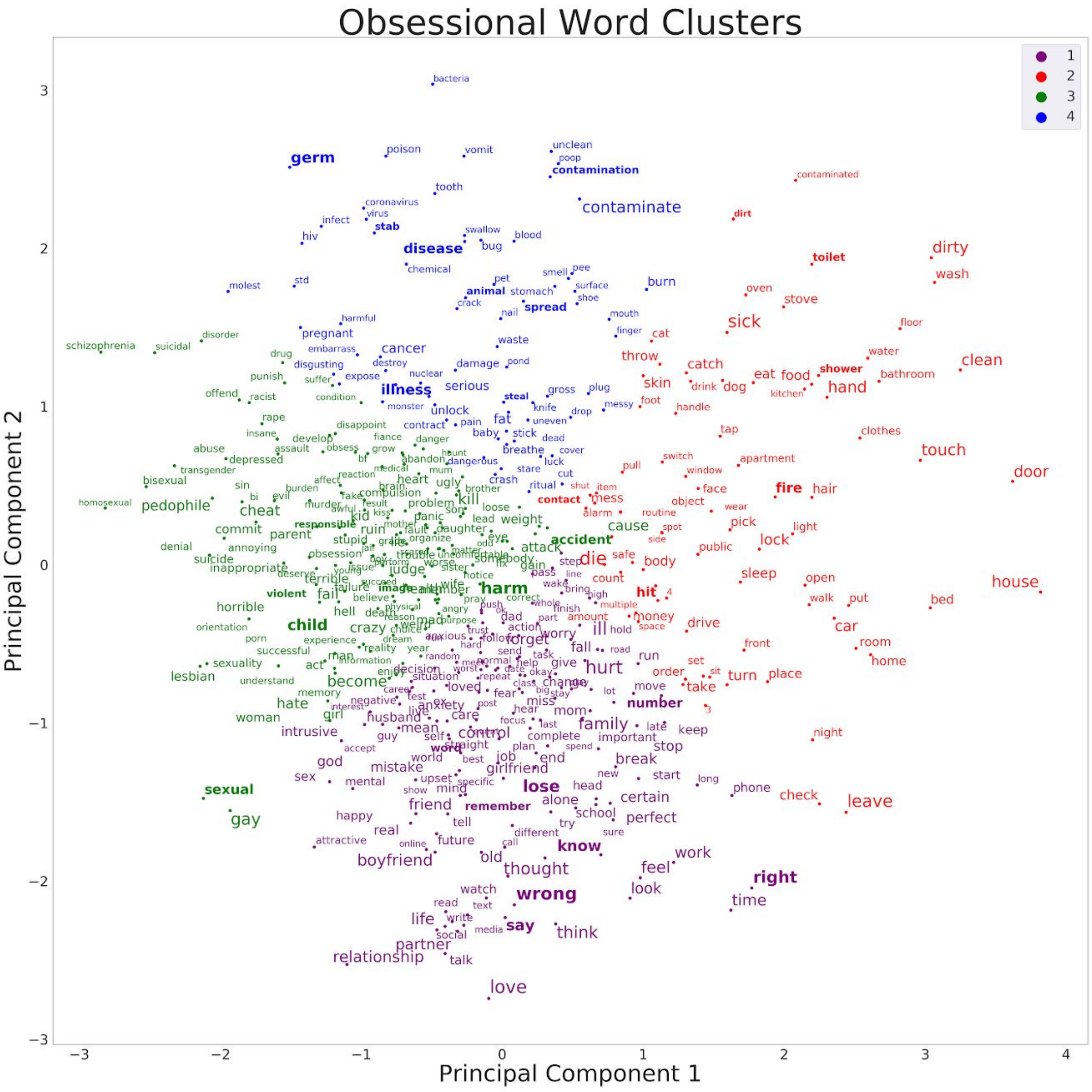

Figure S3. Top 7% most frequently inputted obsessional words. The embedding was trained on the entirety of the dataset, and clustered using k-means with k = 4 clusters. (Note: this is simply a larger version of Fig. 3c.) The font is scaled according to the frequency of occurrence of each word. For reference, **bolded** words are those that also appear in the Yale-Brown Obsessive-Compulsive Scale Symptom Checklist (YBOCS-SC) (Goodman et al. 1989).

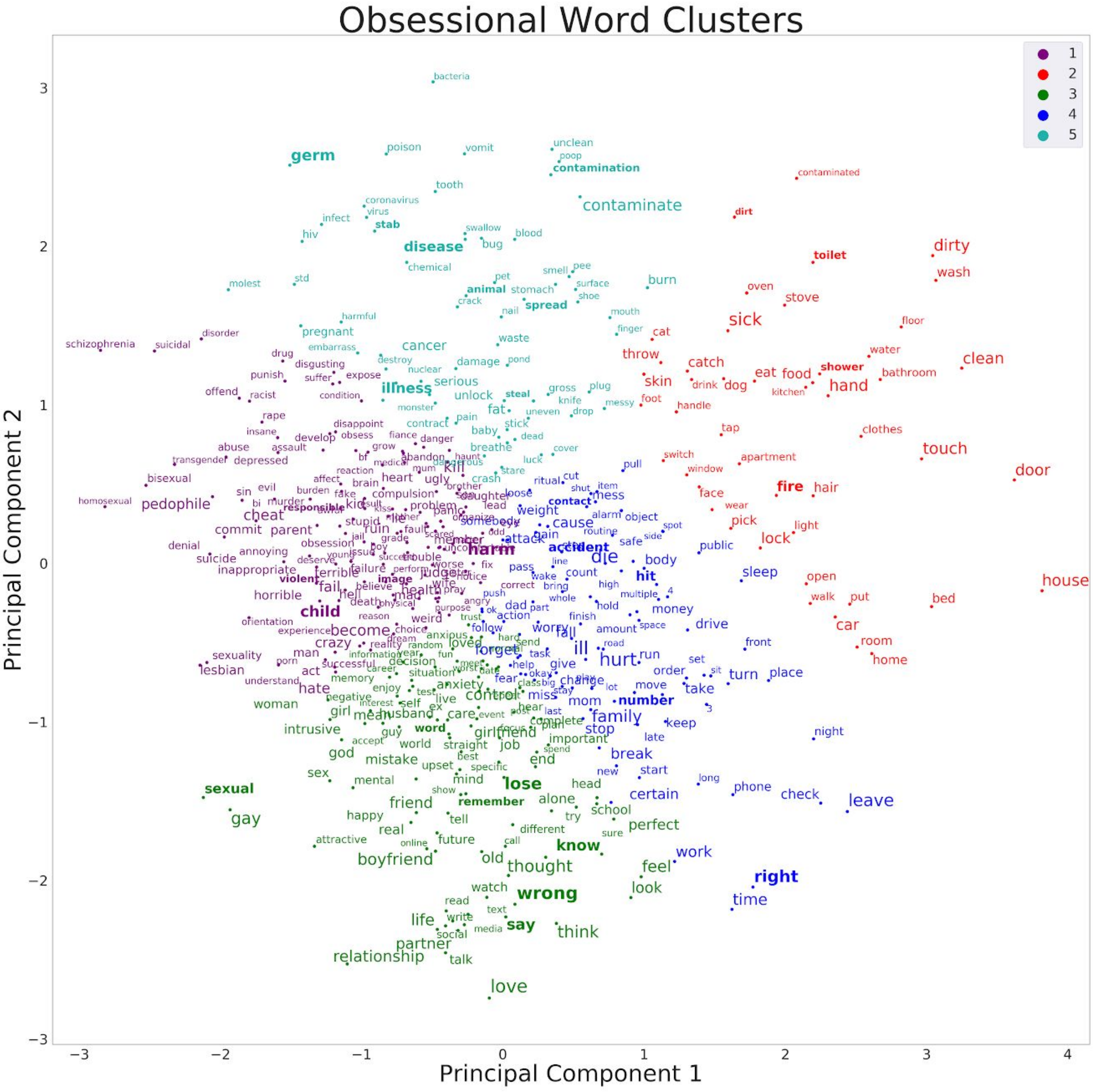

Figure S4. Top 7% most frequently inputted obsessional words. The embedding was trained on the entirety of the dataset, and clustered using k-means with k = 5 clusters. (Note: this is simply a larger version of Fig. 3d.) The font is scaled according to the frequency of occurrence of each

word. For reference, **bolded** words are those that also appear in the Yale-Brown Obsessive-Compulsive Scale Symptom Checklist (YBOCS-SC) (Goodman et al. 1989).

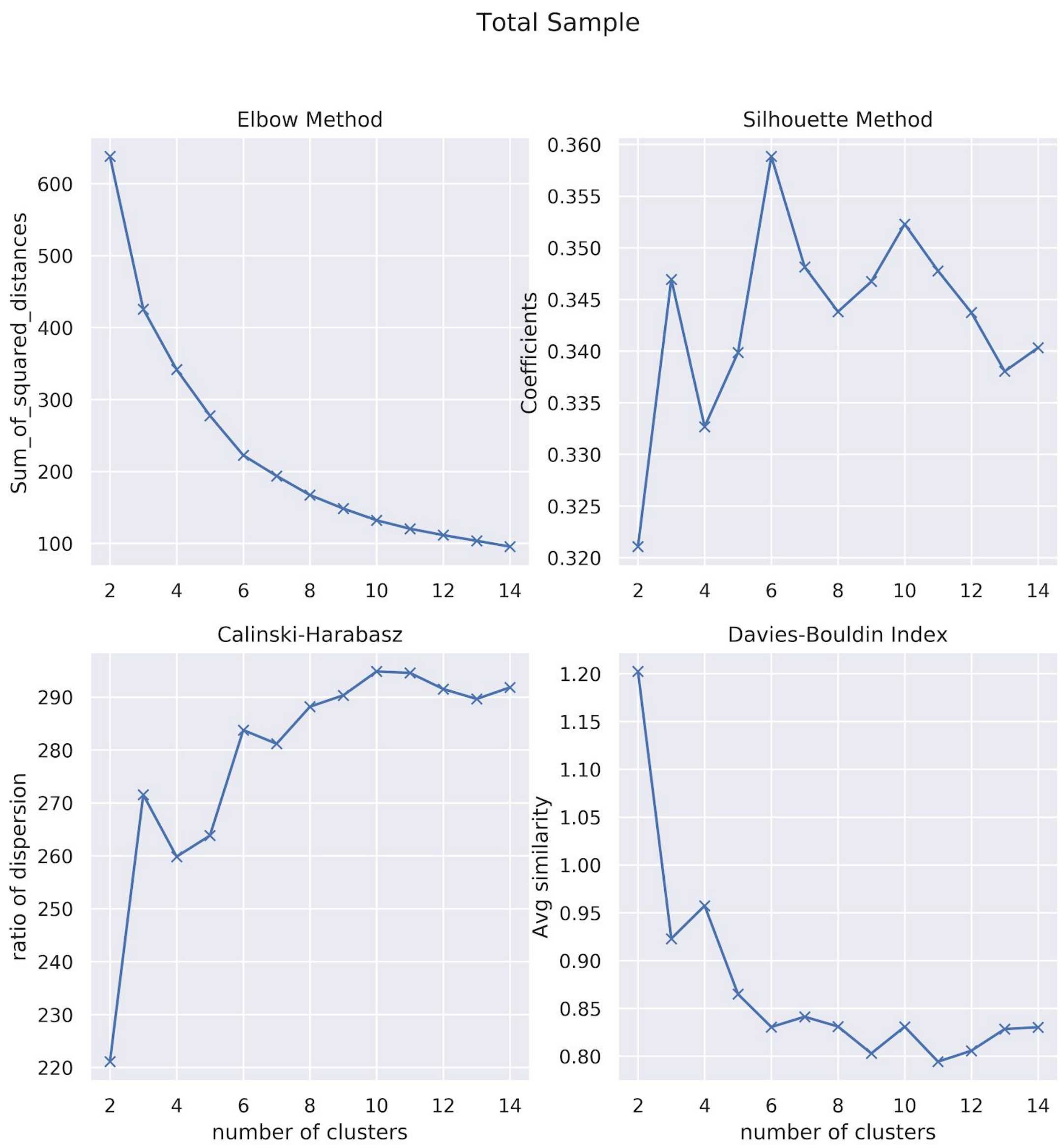

Figure S5. Four heuristic methods to determine optimal number of clusters, calculated on the top 7% most frequently inputted obsessional words. The embedding was trained on the entirety of the dataset and reduced from 100 dimensions to 2 dimensions. The Elbow method's optimal cluster occurs at point with greatest slope change (elbow): k = 3. The Silhouette method's optimal cluster is determined by the highest coefficient score: k = 3. The Calinski-Harabasz method's optimal cluster is determined by the highest score: k = 3. The Davies-Bouldin Index' optimal cluster is determined by lower scores: k = 3.

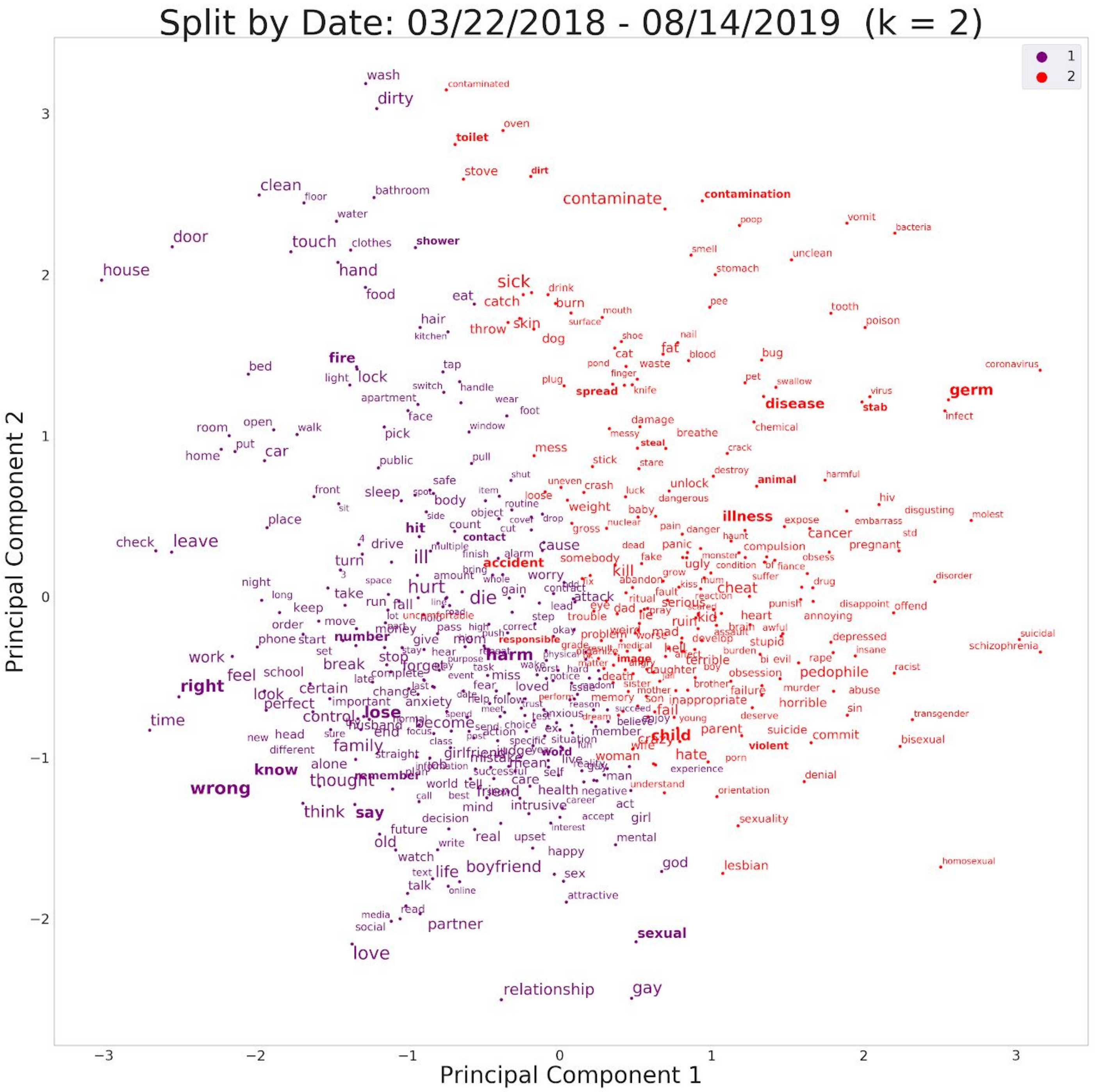

Figure S6. Top 7% most frequently inputted obsessional words. The embedding was trained on all obsessions between March 22, 2018 and August 14, 2019, which represents exactly half of the dataset and is denoted as “Group 1” in the figure title. The data were clustered using k-means with k = 2 clusters. The font is scaled according to the frequency of occurrence of each

word. For reference, **bolded** words are those that also appear in the Yale-Brown Obsessive-Compulsive Scale Symptom Checklist (YBOCS-SC) (Goodman et al. 1989).

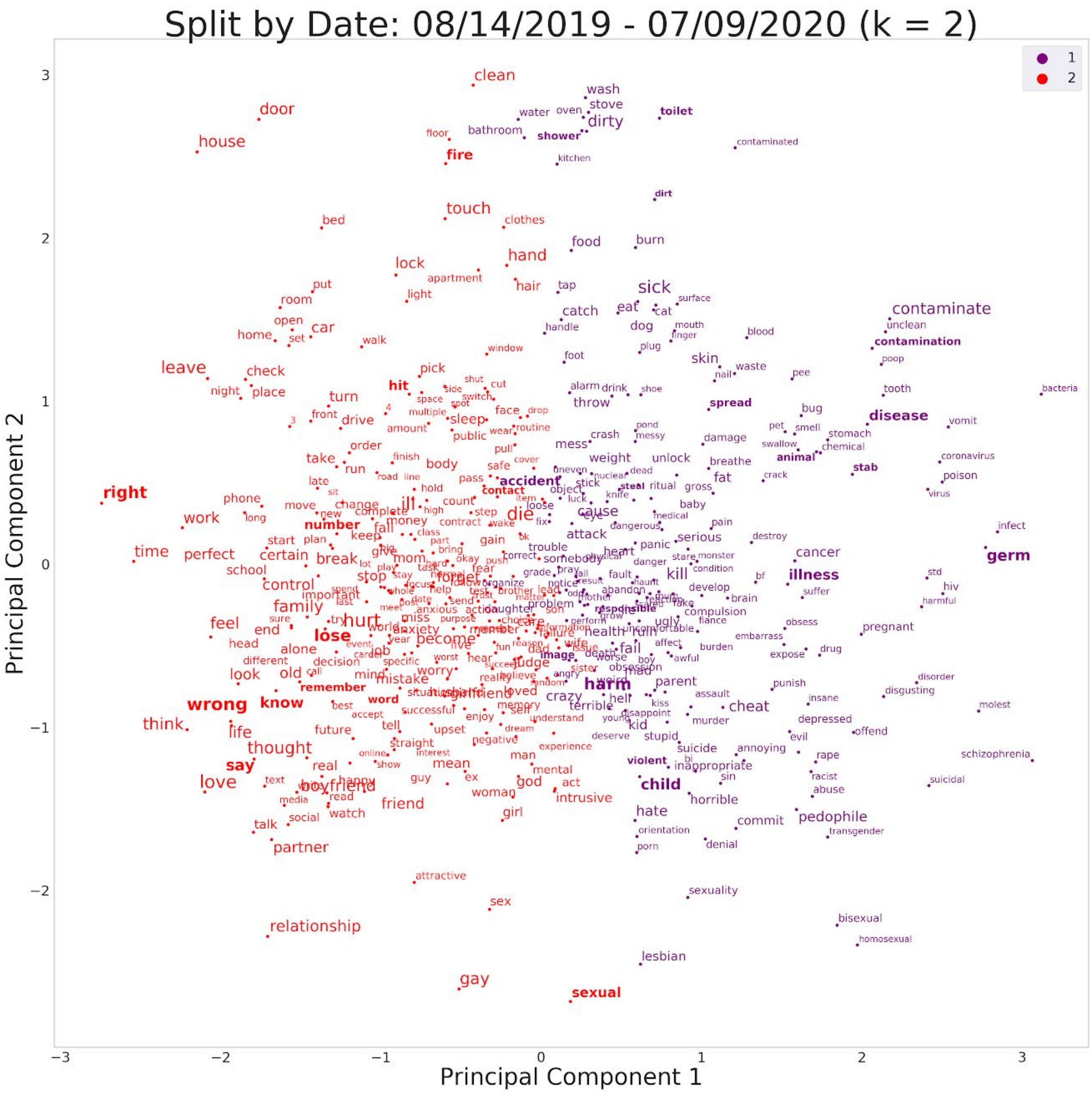

Figure S7. Top 7% most frequently inputted obsessional words. The embedding was trained on all obsessions between August 14, 2019 and July 9, 2020, which represents exactly half of the

dataset and is denoted as “Group 2” in the figure title. The data were clustered using k-means with k = 2 clusters. The font is scaled according to the frequency of occurrence of each word. For reference, **bolded** words are those that also appear in the Yale-Brown Obsessive-Compulsive Scale Symptom Checklist (YBOCS-SC) (Goodman et al. 1989).

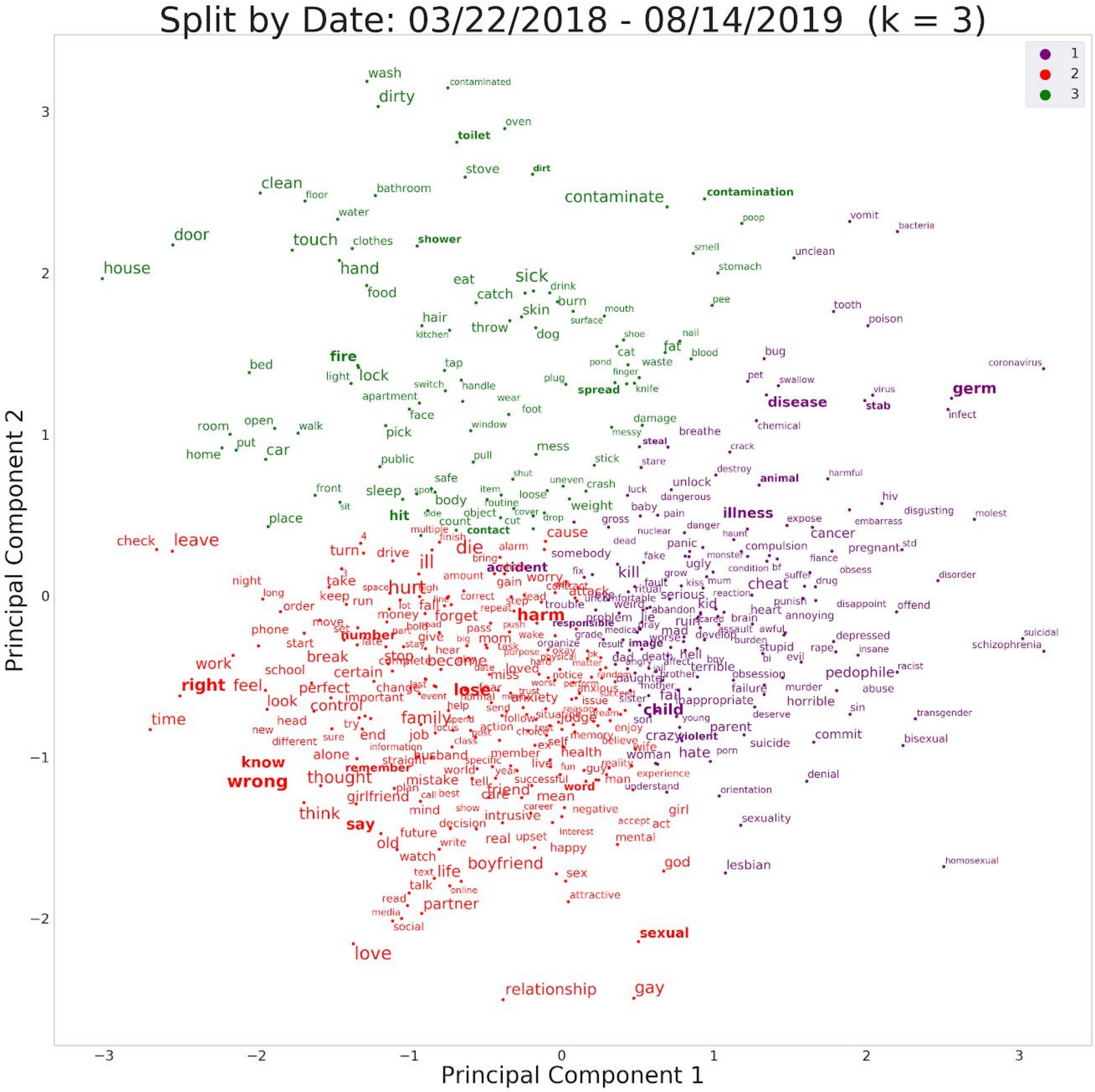

Figure S8. Top 7% most frequently inputted obsessional words. The embedding was trained on all obsessions between March 22, 2018 and August 14, 2019, which represents exactly half of the dataset and is denoted as "Group 1" in the figure title. The data were clustered using k-means with k = 3 clusters. The font is scaled according to the frequency of occurrence of each

word. For reference, **bolded** words are those that also appear in the Yale-Brown Obsessive-Compulsive Scale Symptom Checklist (YBOCS-SC) [(Goodman et al. 1989)](#).

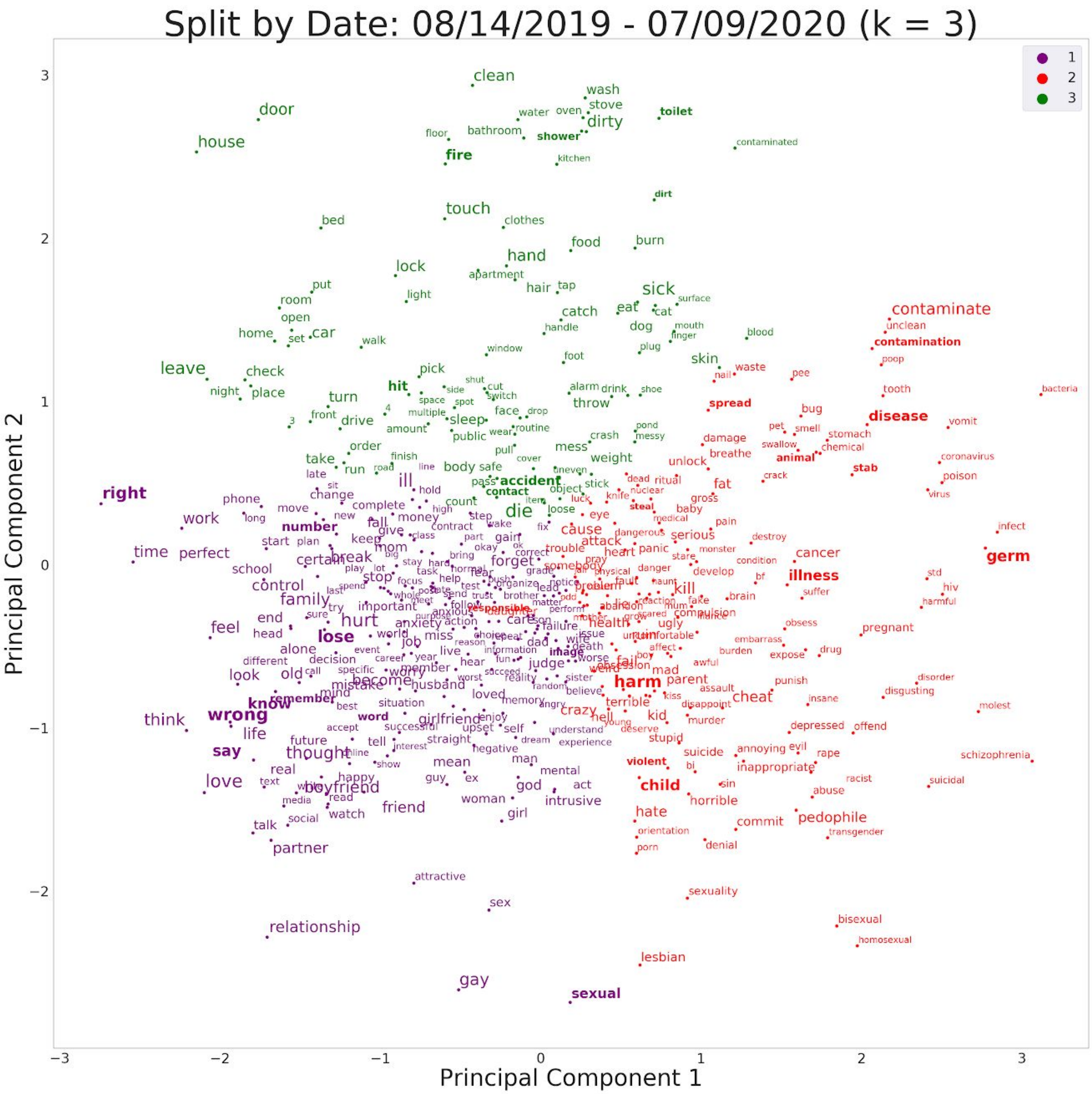

Figure S9. Top 7% most frequently inputted obsessional words. The embedding was trained on all obsessions between August 14, 2019 and July 9, 2020, which represents exactly half of the

dataset and is denoted as “Group 2” in the figure title. The data were clustered using k-means with k = 3 clusters. The font is scaled according to the frequency of occurrence of each word. For reference, **bolded** words are those that also appear in the Yale-Brown Obsessive-Compulsive Scale Symptom Checklist (YBOCS-SC) (Goodman et al. 1989).

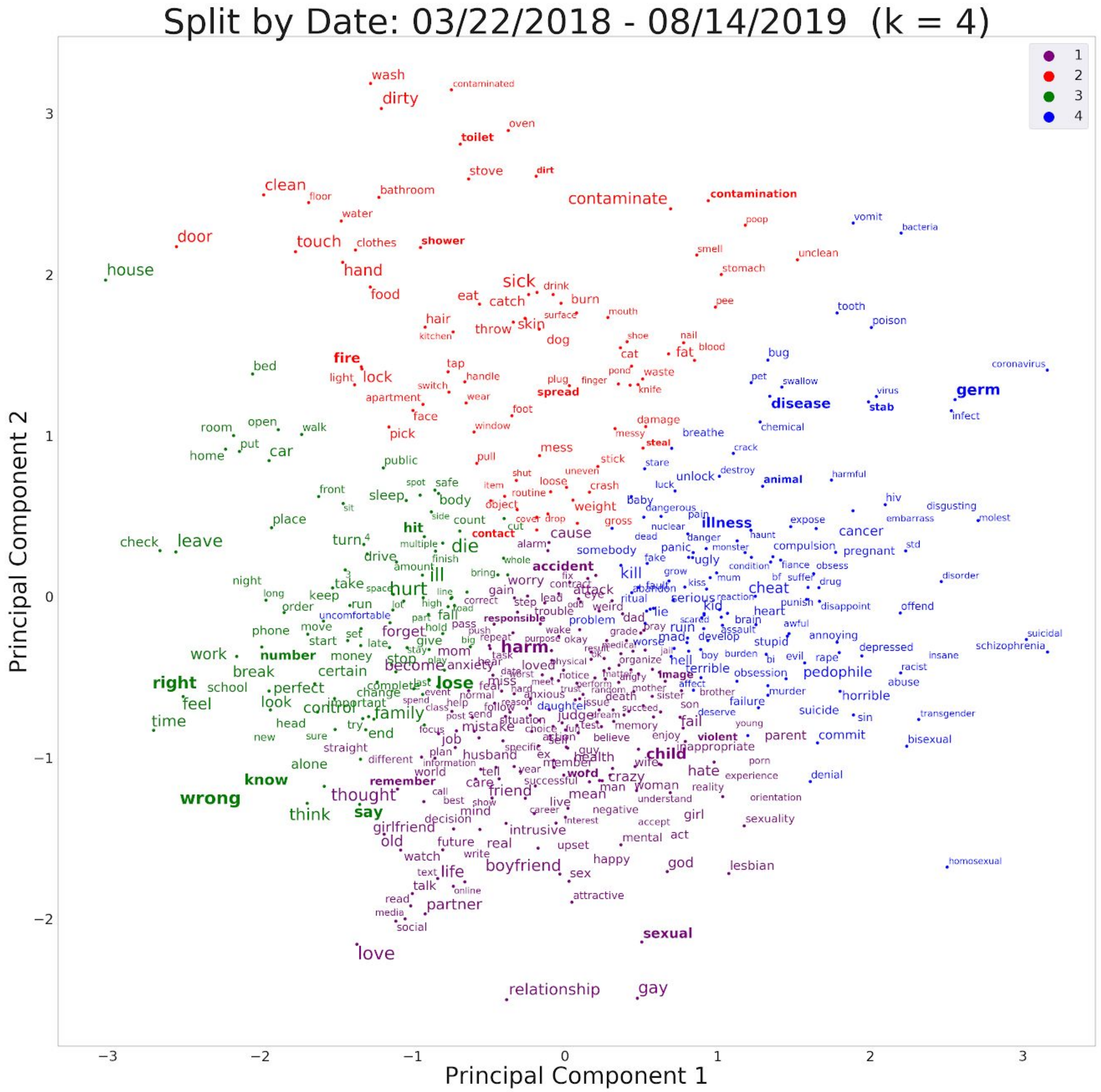

Figure S10. Top 7% most frequently inputted obsessional words. The embedding was trained on all obsessions between March 22, 2018 and August 14, 2019, which represents exactly half of the dataset and is denoted as “Group 1” in the figure title. The data were clustered using k-means with k = 4 clusters. The font is scaled according to the frequency of occurrence of each

word. For reference, **bolded** words are those that also appear in the Yale-Brown Obsessive-Compulsive Scale Symptom Checklist (YBOCS-SC) (Goodman et al. 1989).

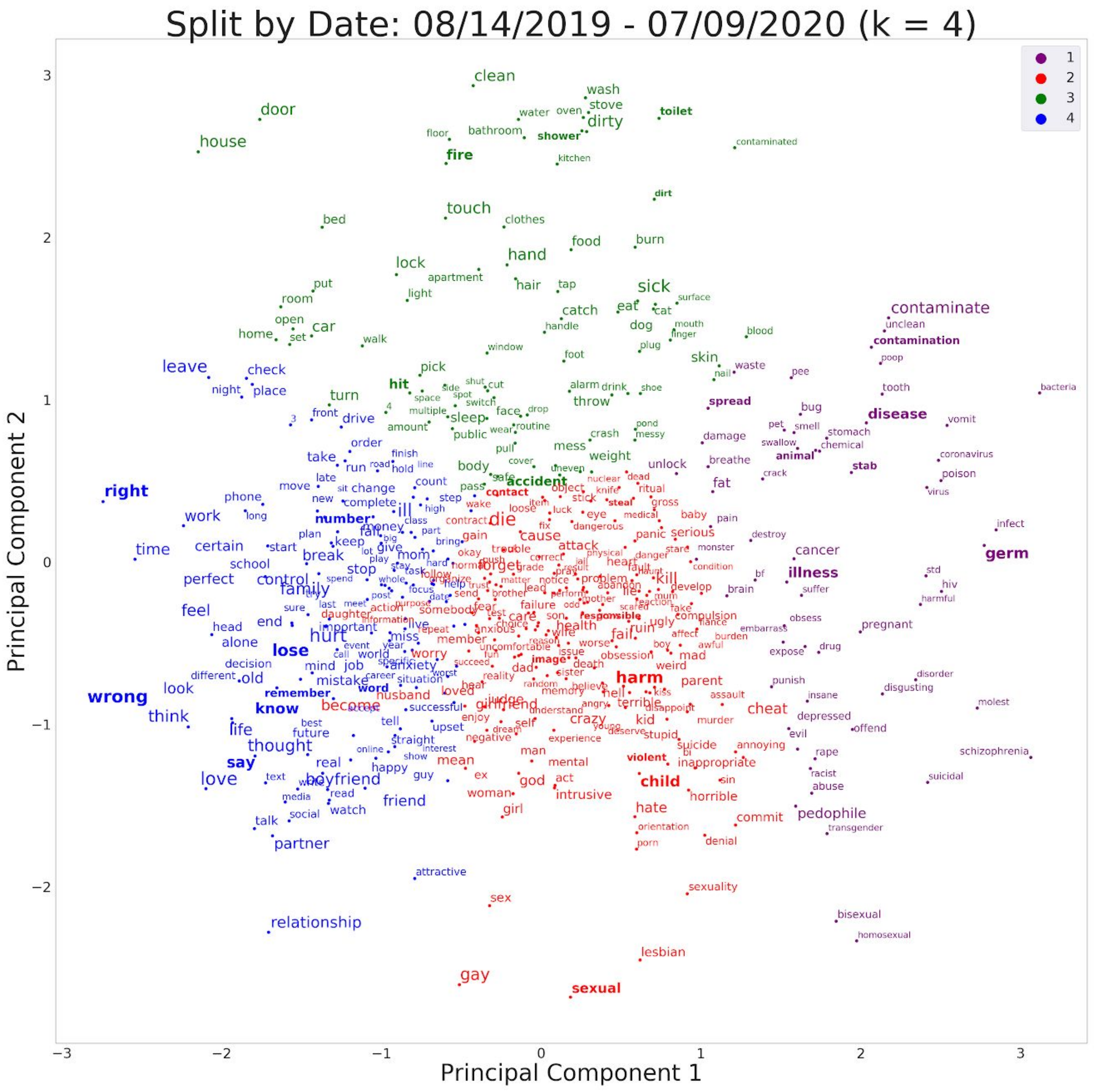

Figure S11. Top 7% most frequently inputted obsessional words. The embedding was trained on all obsessions between August 14, 2019 and July 9, 2020, which represents exactly half of the

dataset and is denoted as “Group 2” in the figure title. The data were clustered using k-means with k = 4 clusters. The font is scaled according to the frequency of occurrence of each word. For reference, **bolded** words are those that also appear in the Yale-Brown Obsessive-Compulsive Scale Symptom Checklist (YBOCS-SC) (Goodman et al. 1989).

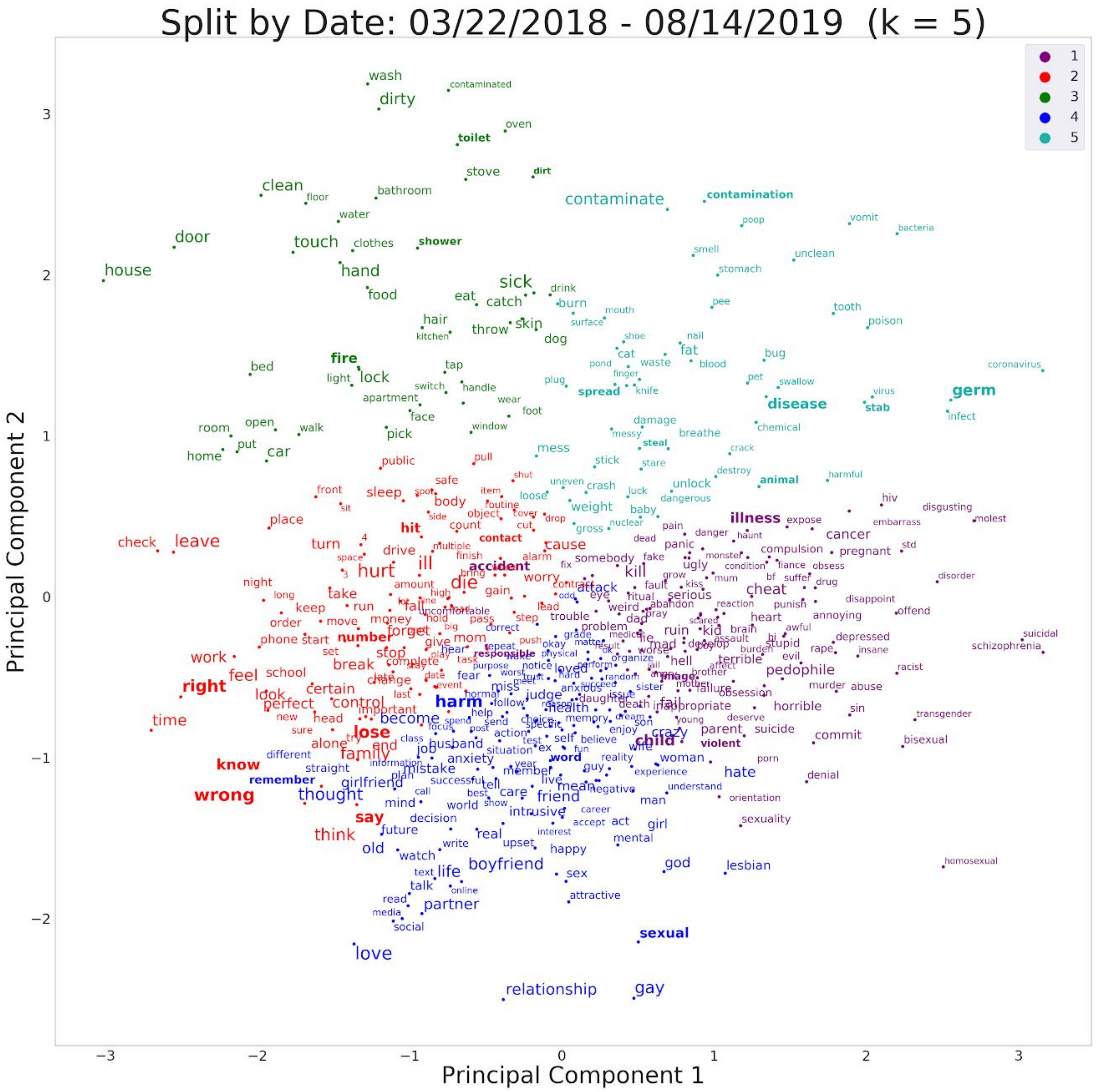

Figure S12. Top 7% most frequently inputted obsessional words. The embedding was trained on all obsessions between March 22, 2018 and August 14, 2019, which represents exactly half of the dataset and is denoted as “Group 1” in the figure title. The data were clustered using k-means with k = 5 clusters. The font is scaled according to the frequency of occurrence of each

word. For reference, **bolded** words are those that also appear in the Yale-Brown Obsessive-Compulsive Scale Symptom Checklist (YBOCS-SC) (Goodman et al. 1989).

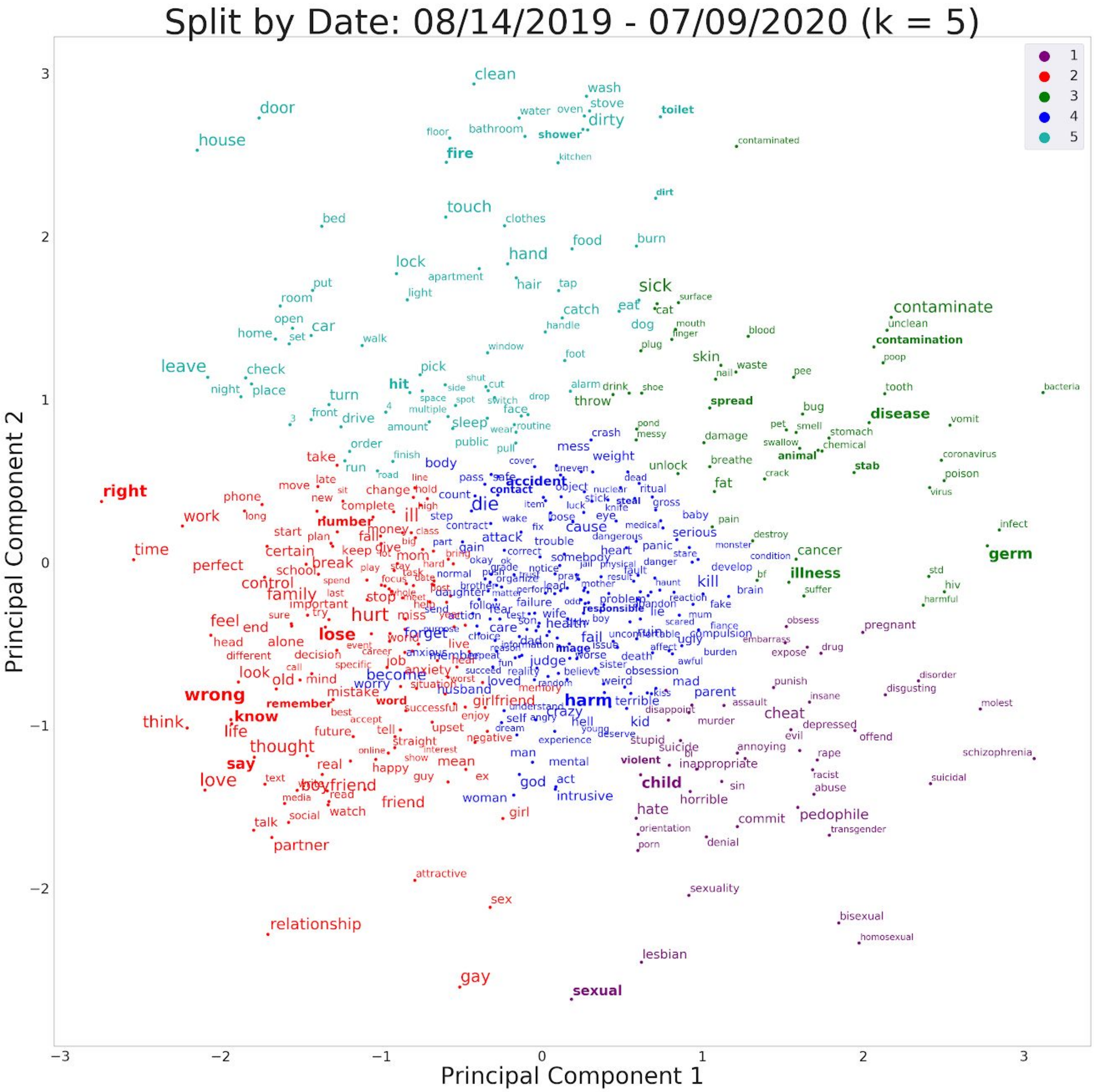

Figure S13. Top 7% most frequently inputted obsessional words. The embedding was trained on all obsessions between August 14, 2019 and July 9, 2020, which represents exactly half of the

dataset and is denoted as “Group 2” in the figure title. The data were clustered using k-means with k = 5 clusters. The font is scaled according to the frequency of occurrence of each word. For reference, **bolded** words are those that also appear in the Yale-Brown Obsessive-Compulsive Scale Symptom Checklist (YBOCS-SC) (Goodman et al. 1989).

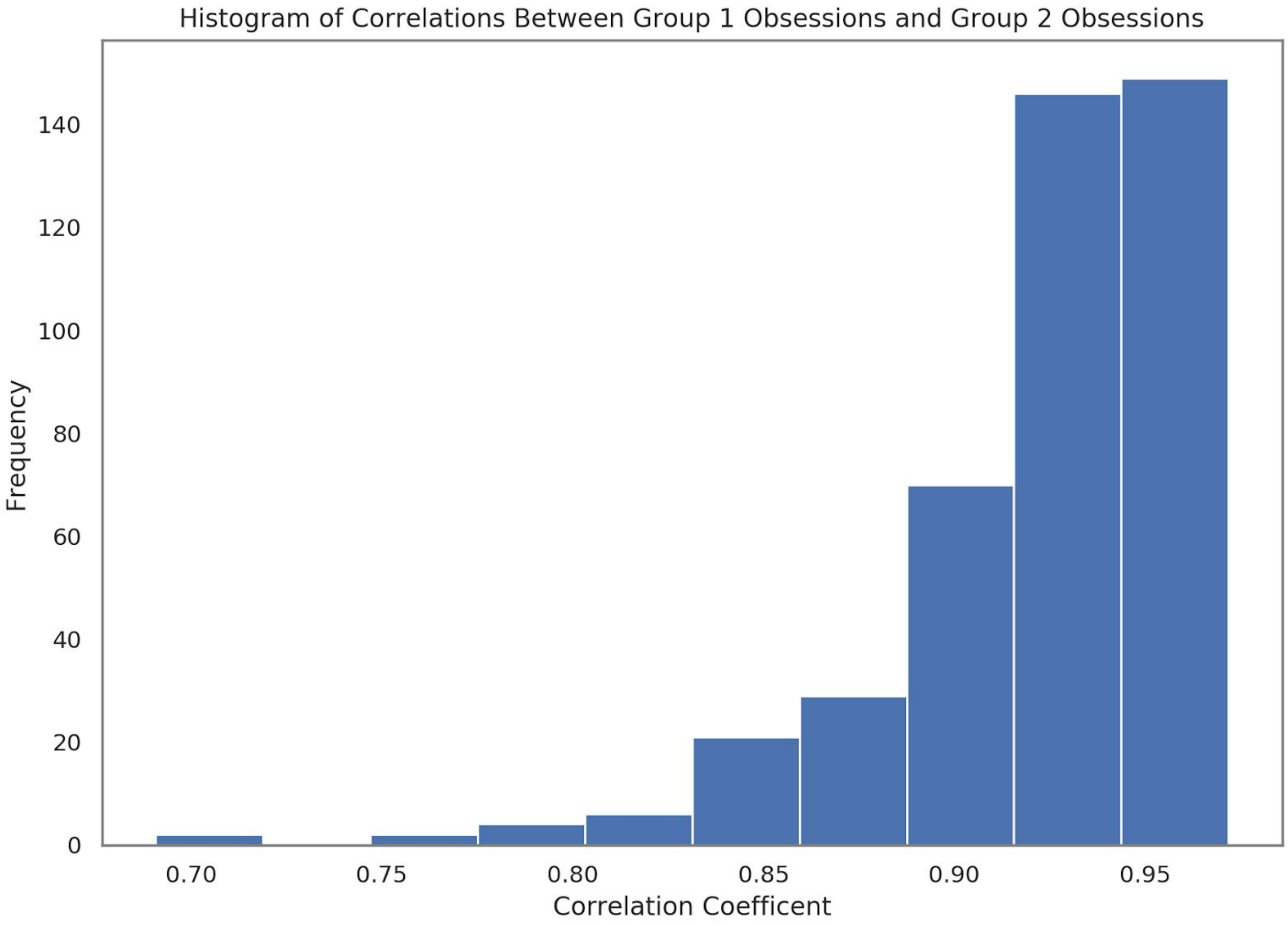

Figure S14. Histogram of Pearson correlation coefficient between obsessions of Group 1 and obsessions of Group 2 split samples by non-overlapping dates. Two-dimensional embeddings of the two groups were compared by calculating the distance from a word to all other words on the graph and done for all words creating a matrix of distances. Matrices for the two groups were compared using the Pearson correlation coefficient. The majority of correlations were high, demonstrating high consistency of results across separate time periods.

Figure S15a. Four heuristic methods on obsessions between 03/22/2018 and 08/14/2019.

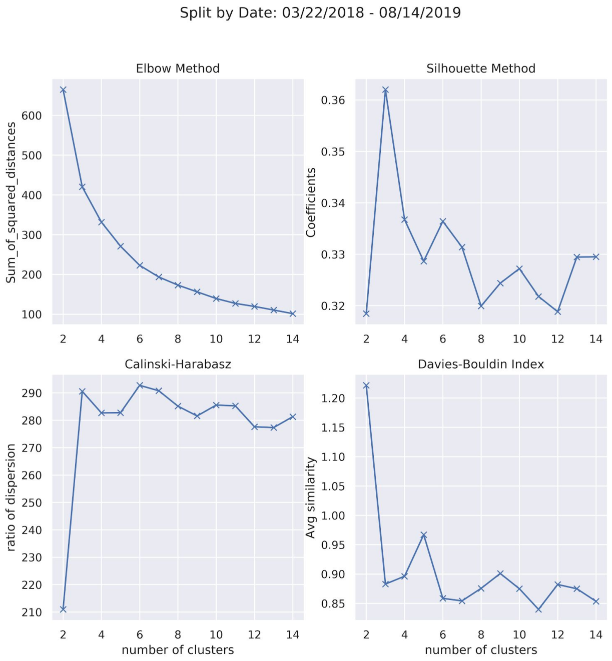

Figure S15b. Four heuristic methods on obsessions between 08/14/2019 and 07/09/2020.

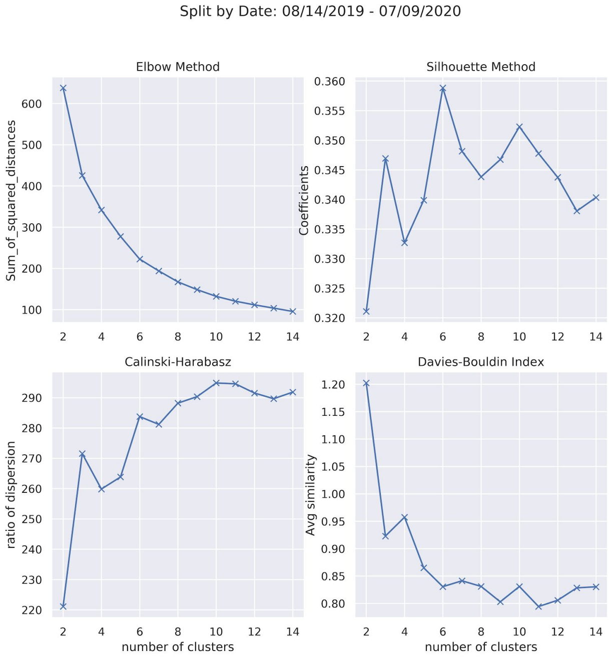

Figure S15. Four heuristic methods to determine optimal number of clusters. Calculated on top 7% most frequently inputted obsessional words and for each split-sample by non-overlapping date "Group." The embeddings were trained on respective groups and reduced from 100 dimensions to 2 dimensions. The Elbow method's optimal cluster for both groups occurs at point with greatest slope change (elbow): k = 3. The Silhouette method's optimal cluster for both groups is determined by the highest coefficient score: k = 3. The Calinski-Harabasz method's optimal cluster is determined by the highest score: k = 3. The Davies-Bouldin Index' optimal cluster is determined by lower scores: k = 3.

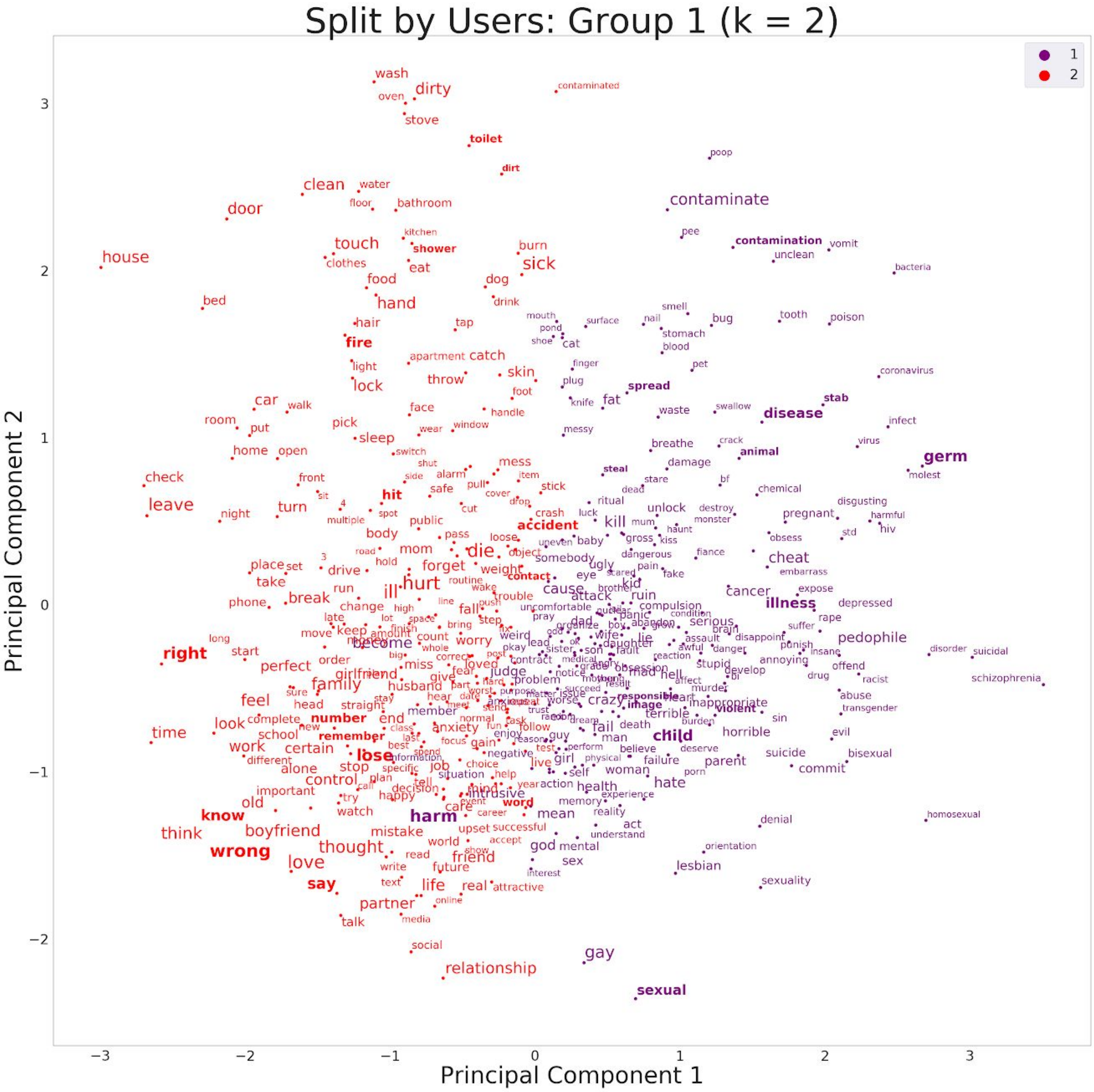

Figure S16. Top 7% most frequently inputted obsessional words. The embedding was trained on “Group 1” of users, which represents a randomly sampled half of the users. Data were clustered using k-means with k = 2 clusters. The font is scaled according to the frequency of occurrence of each word. For reference, **bolded** words are those that also appear in the Yale-Brown Obsessive-Compulsive Scale Symptom Checklist (YBOCS-SC) (Goodman et al. 1989).

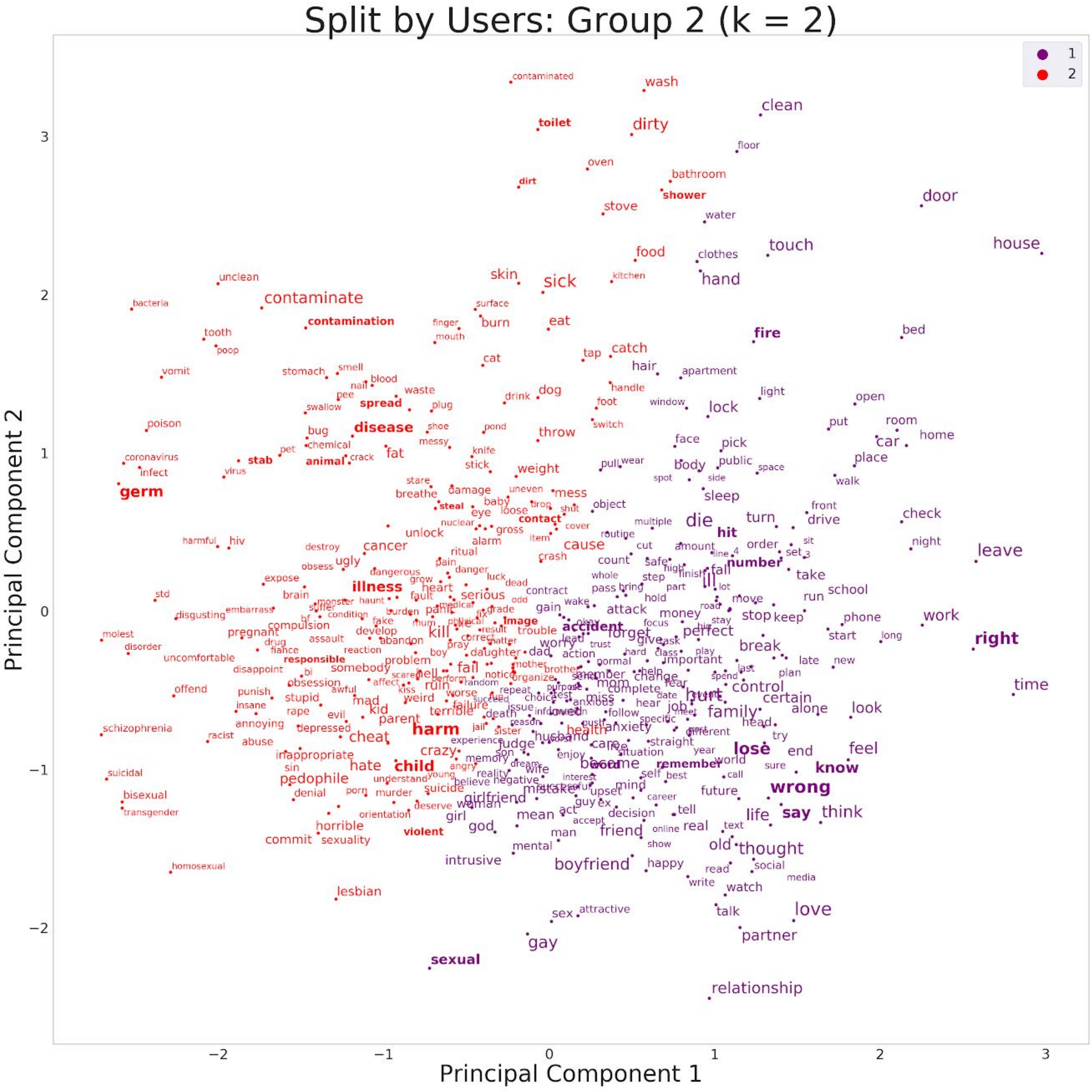

Figure S17. Top 7% most frequently inputted obsessional words. The embedding was trained on “Group 2” of users, which represents a randomly sampled half of users. Data were clustered using k-means with k = 2 clusters. The font is scaled according to the frequency of occurrence of

each word. For reference, **bolded** words are those that also appear in the Yale-Brown Obsessive-Compulsive Scale Symptom Checklist (YBOCS-SC) [(Goodman et al. 1989)](#).

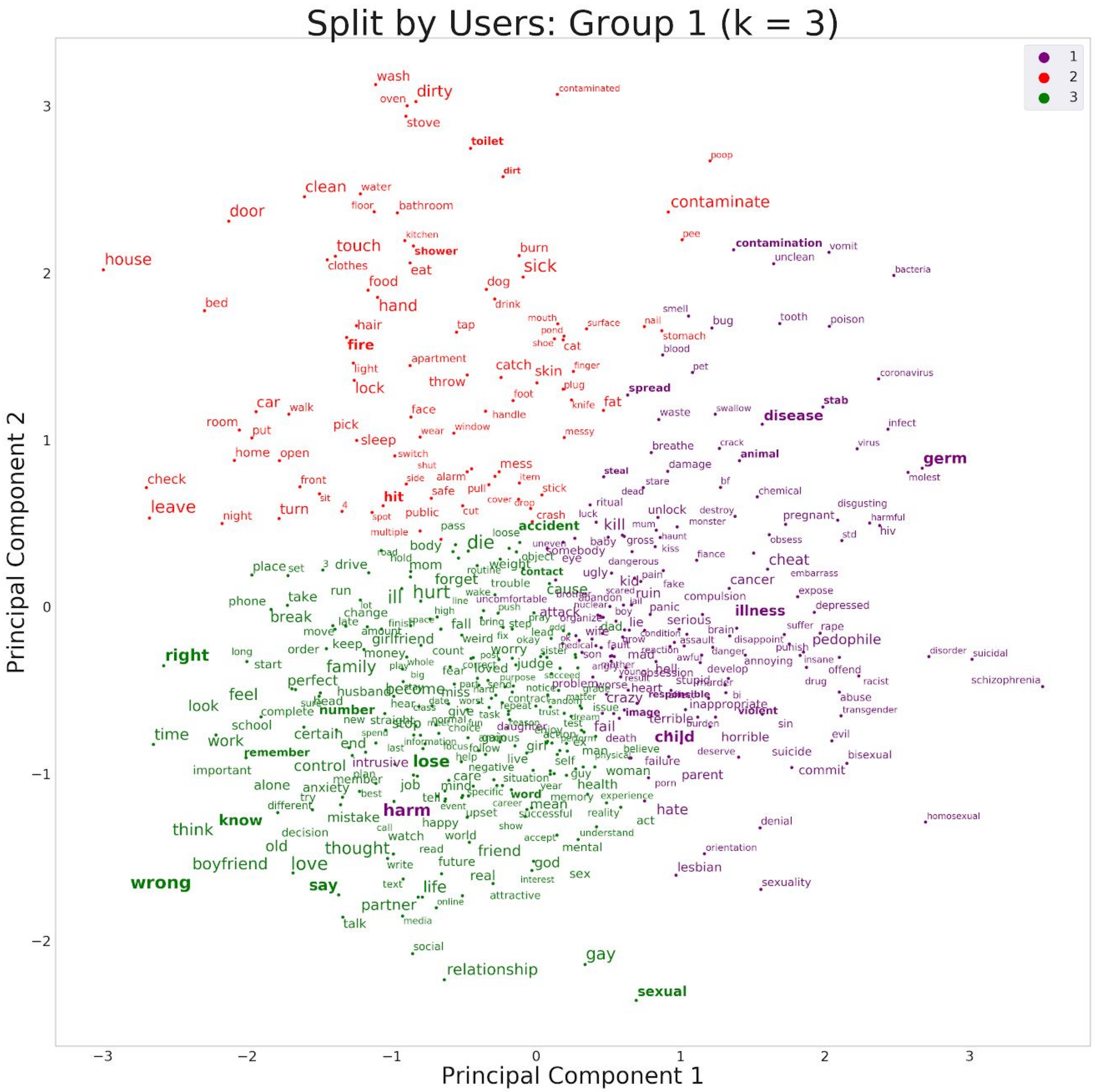

Figure S18. Top 7% most frequently inputted obsessional words. The embedding was trained on “Group 1” of users, which represents a randomly sampled half of the users. Data were clustered

using k-means with k = 3 clusters. The font is scaled according to the frequency of occurrence of each word. For reference, **bolded** words are those that also appear in the Yale-Brown Obsessive-Compulsive Scale Symptom Checklist (YBOCS-SC) (Goodman et al. 1989).

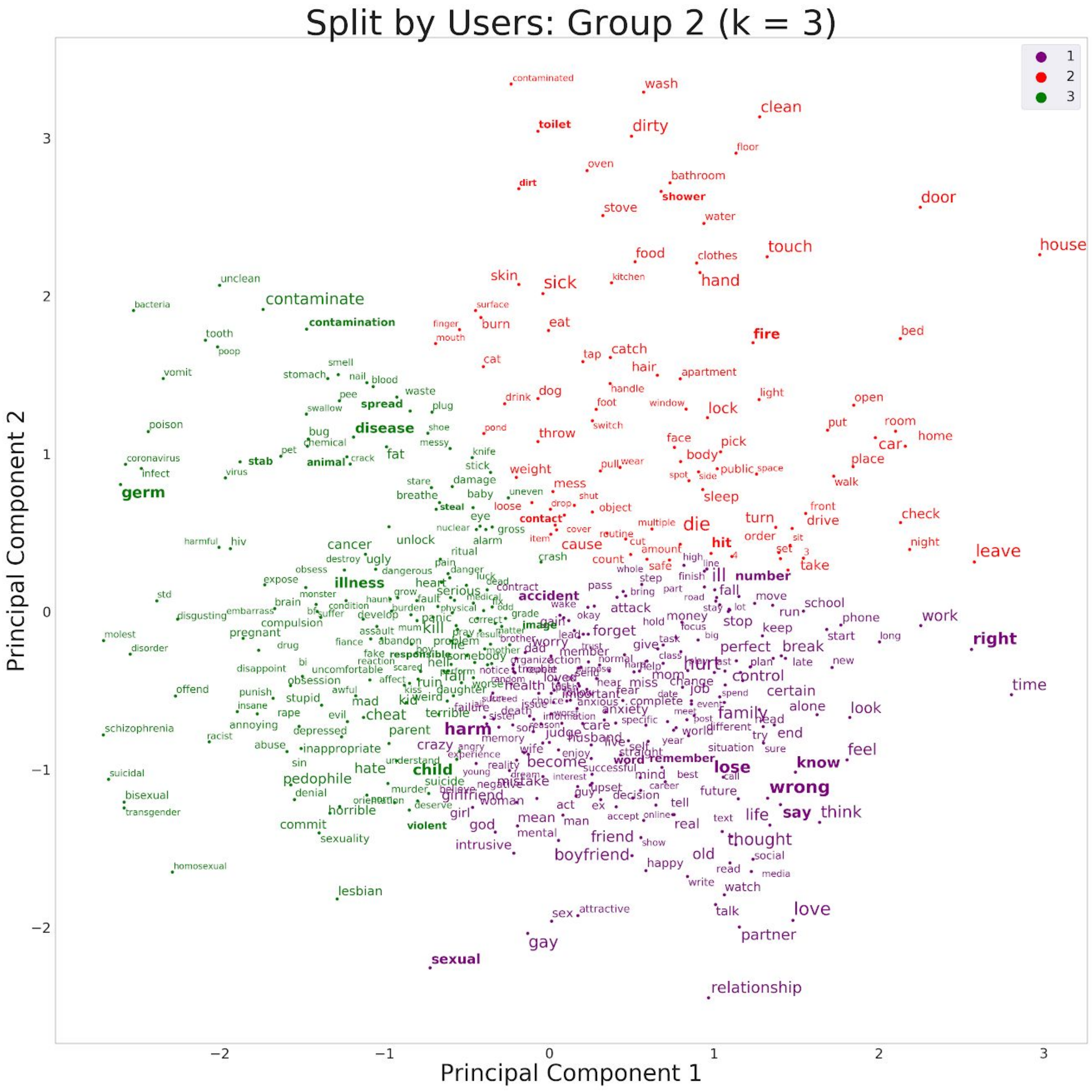

Figure S19. Top 7% most frequently inputted obsessional words. The embedding was trained on “Group 2” of users, which represents a randomly sampled half of the users. Data were clustered using k-means with k = 3 clusters. The font is scaled according to the frequency of occurrence of each word. For reference, **bolded** words are those that also appear in the Yale-Brown Obsessive-Compulsive Scale Symptom Checklist (YBOCS-SC) (Goodman et al. 1989).

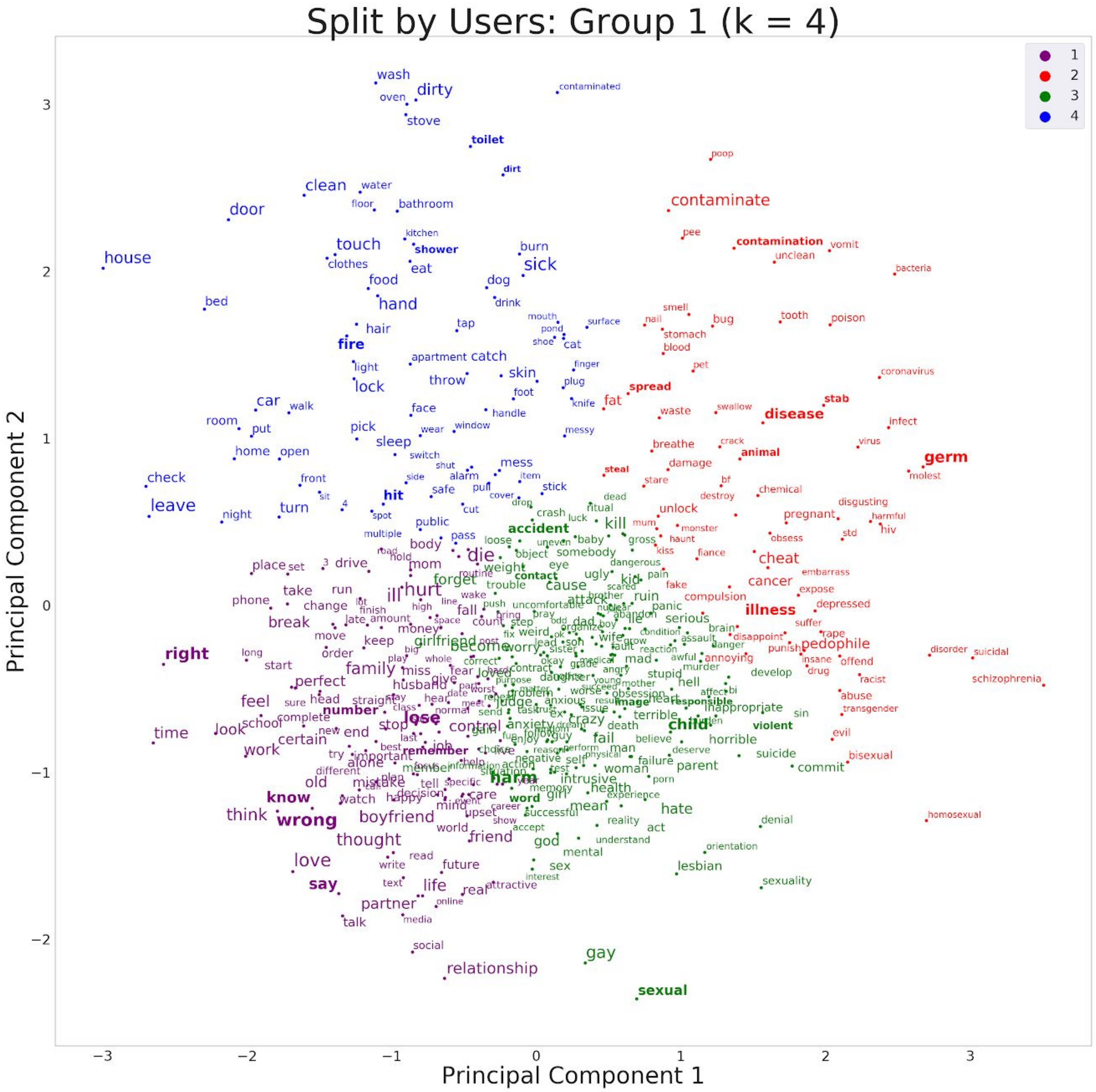

Figure S20. Top 7% most frequently inputted obsessional words. The embedding was trained on “Group 1” of users, which represents a randomly sampled half of the users. Data were clustered using k-means with k = 4 clusters. The font is scaled according to the frequency of occurrence of

each word. For reference, **bolded** words are those that also appear in the Yale-Brown Obsessive-Compulsive Scale Symptom Checklist (YBOCS-SC) (Goodman et al. 1989).

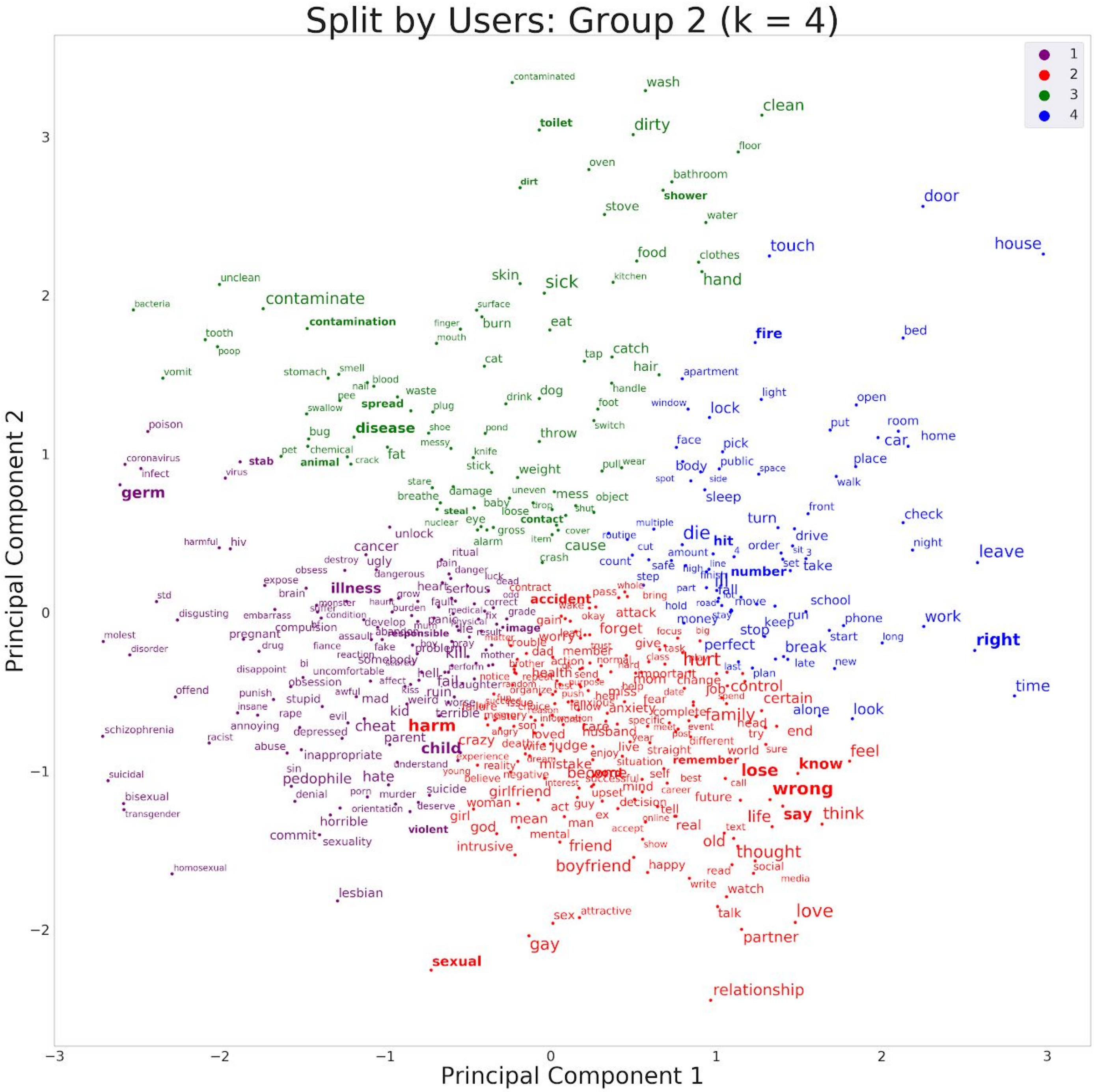

Figure S21. Top 7% most frequently inputted obsessional words. The embedding was trained on "Group 2" of users, which represents a randomly sampled half of the users. Data were clustered using k-means with k = 4 clusters. The font is scaled according to the frequency of occurrence of

each word. For reference, **bolded** words are those that also appear in the Yale-Brown Obsessive-Compulsive Scale Symptom Checklist (YBOCS-SC) (Goodman et al. 1989).

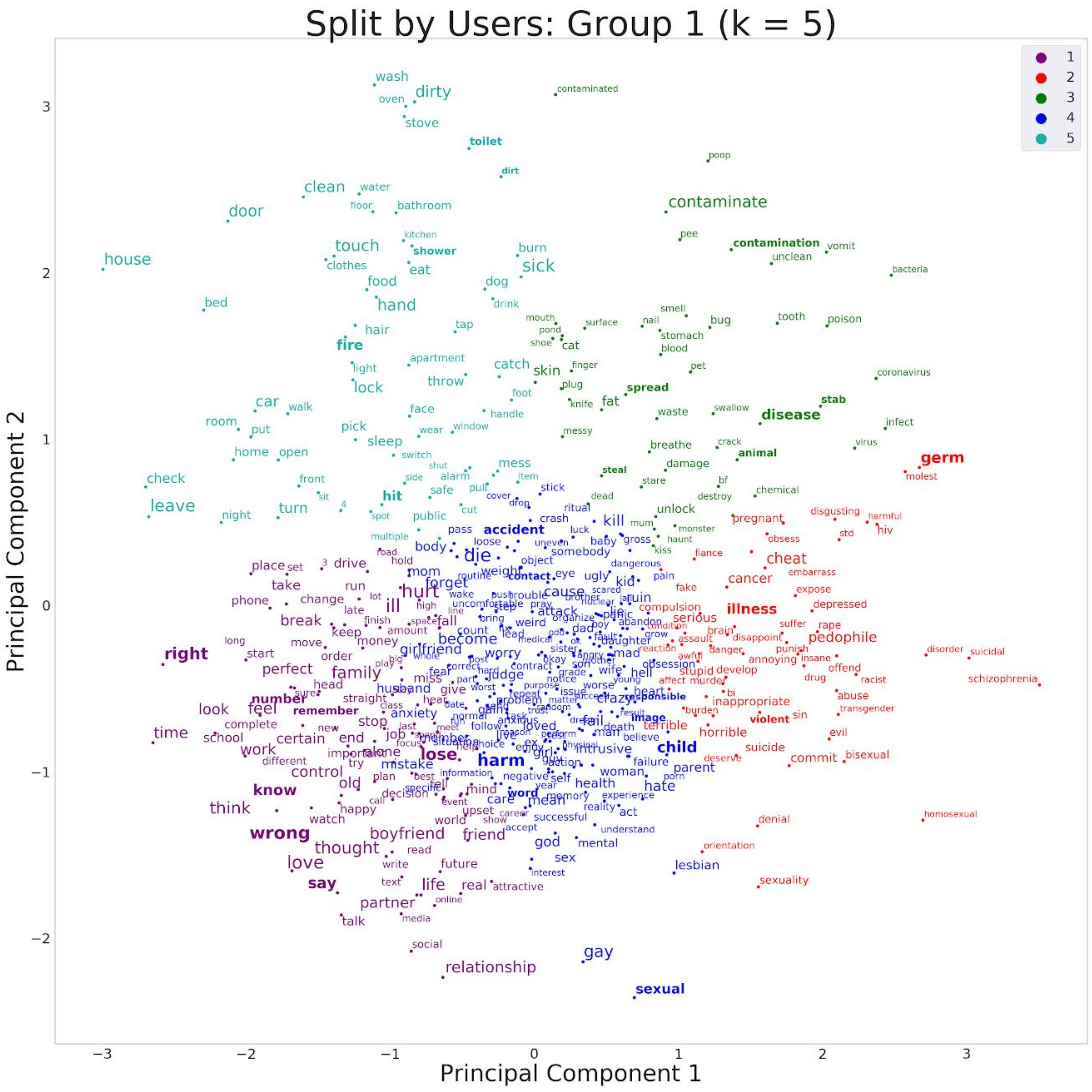

Figure S22. Top 7% most frequently inputted obsessional words. The embedding was trained on “Group 1” of users, which represents a randomly sampled half of the users. Data were clustered using k-means with k = 5 clusters. The font is scaled according to the frequency of occurrence of

each word. For reference, **bolded** words are those that also appear in the Yale-Brown Obsessive-Compulsive Scale Symptom Checklist (YBOCS-SC) [(Goodman et al. 1989)]().

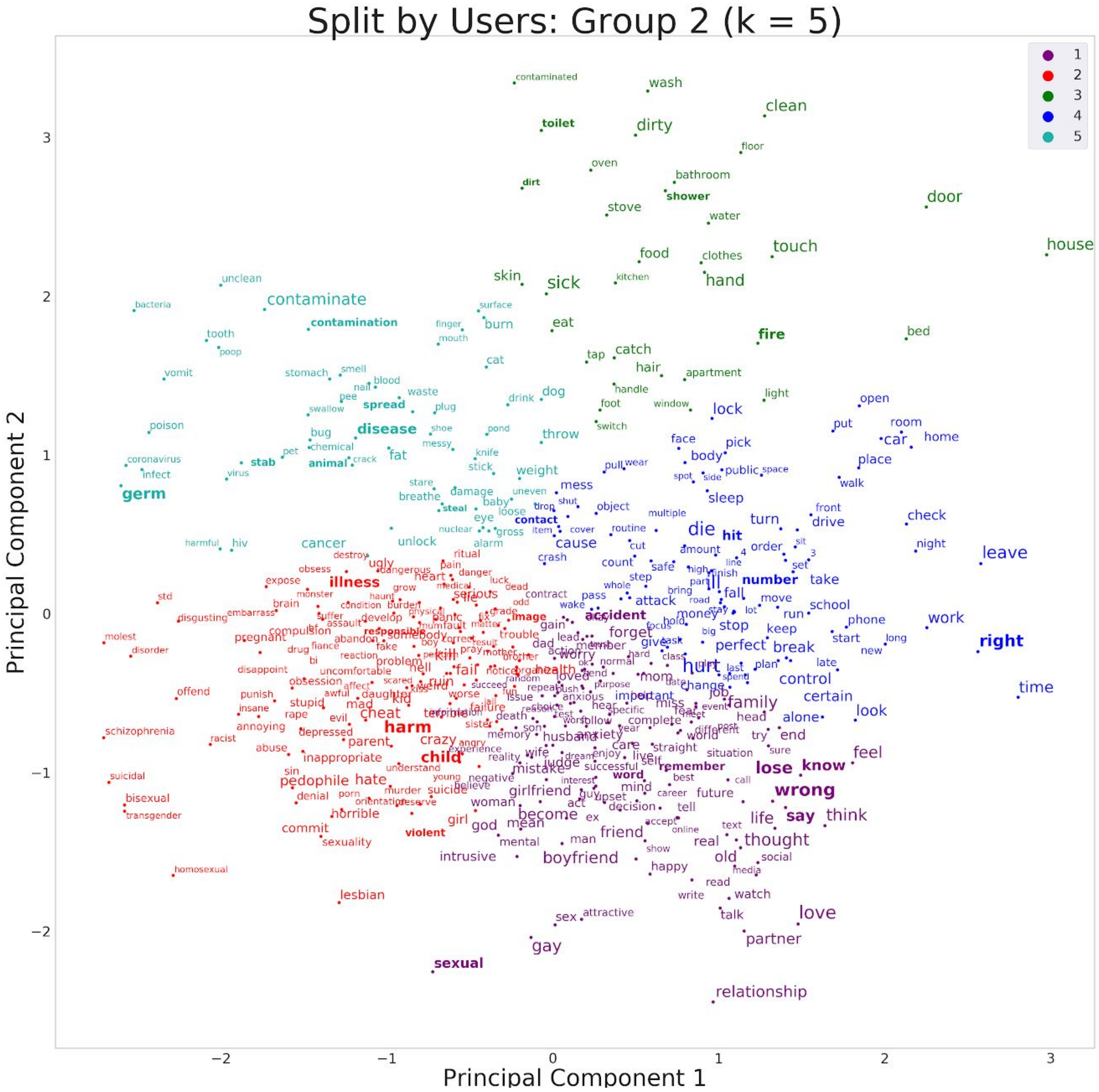

Figure S23. Top 7% most frequently inputted obsessional words. The embedding was trained on “Group 2” of users, which represents a randomly sampled half of the users. Data were clustered using k-means with k = 5 clusters. The font is scaled according to the frequency of occurrence of each word. For reference, **bolded** words are those that also appear in the Yale-Brown Obsessive-Compulsive Scale Symptom Checklist (YBOCS-SC) [(Goodman et al. 1989)]().

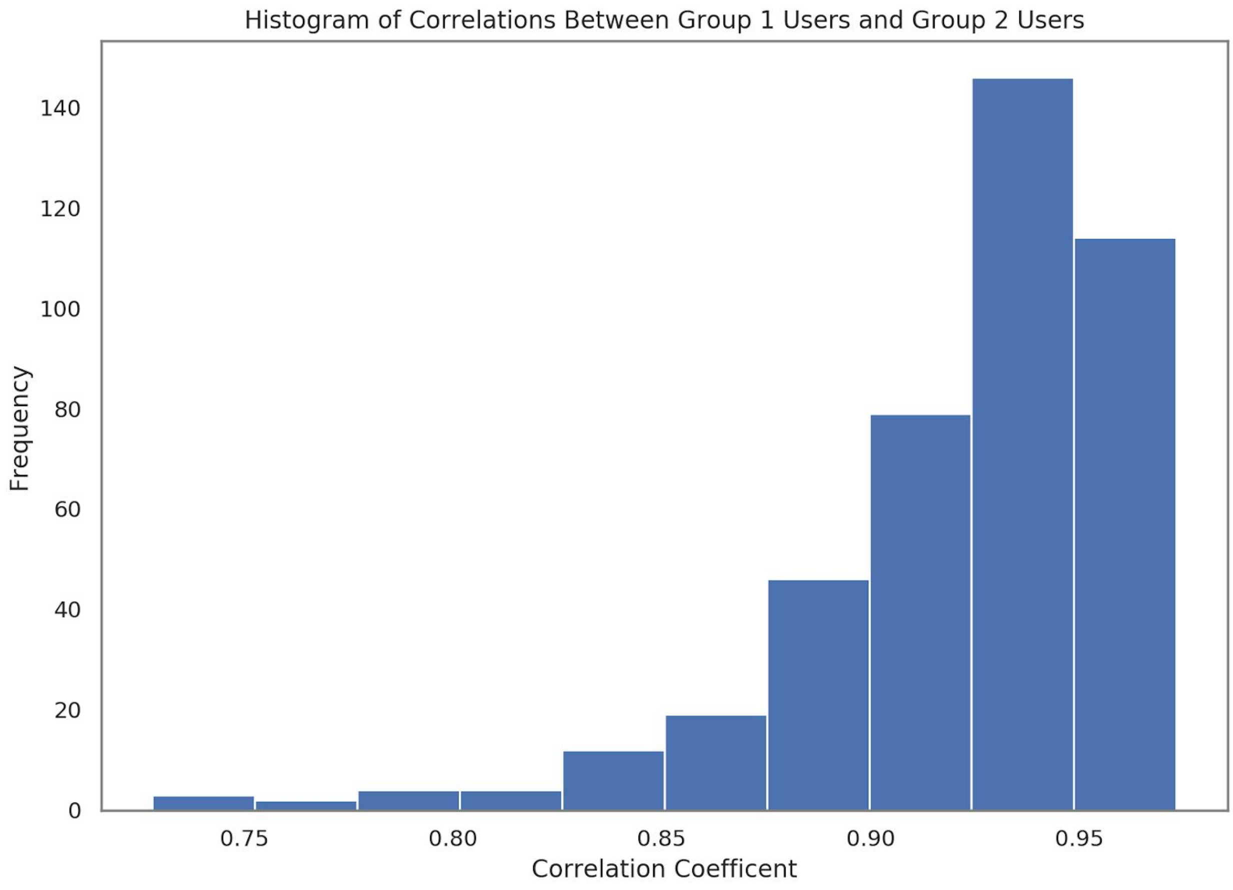

Figure S24. Histogram of Pearson correlation coefficient between the split sample of obsessions of Group 1 of users and obsessions of Group 2 of users. Two-dimensional embeddings of the two groups were compared by calculating the distance from a word to all other words on the graph and done for all words creating a matrix of distances. Matrices for the two groups were compared using the Pearson correlation coefficient. The majority of correlations were high, demonstrating high consistency of results across two sets of separate, randomly-sampled users.

Figure S25a. Four heuristic methods on group 1 of users.

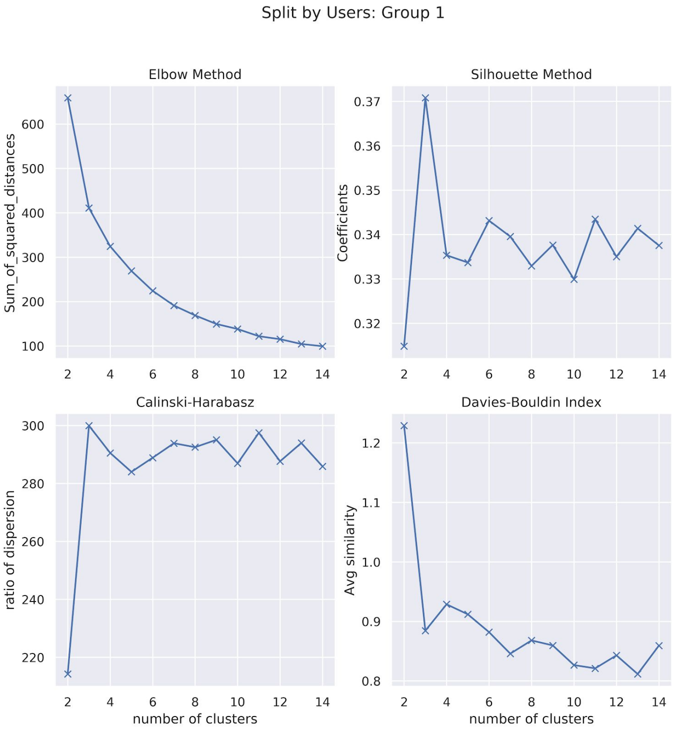

Figure s25b. Four heuristic methods on group 2 of users.

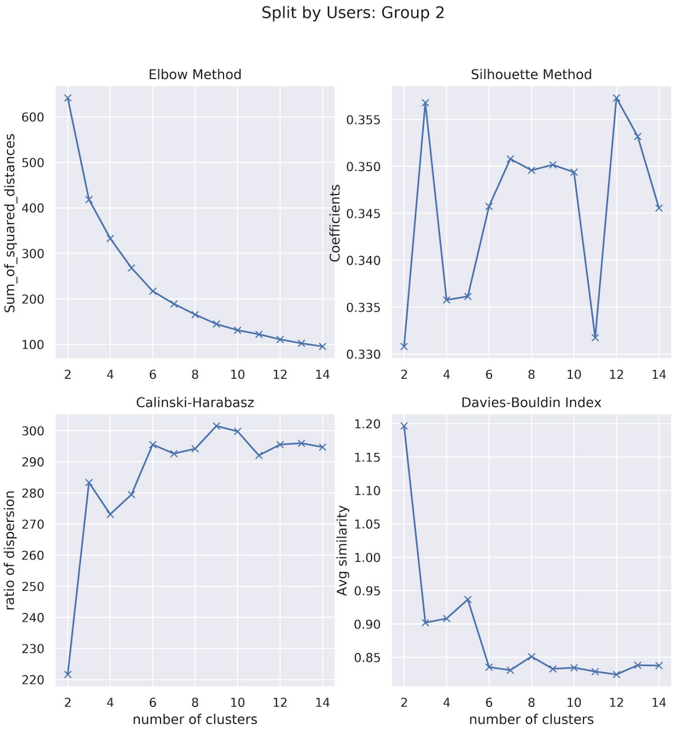

Figure S25. Four heuristic methods to determine optimal number of clusters. Calculated on the top 7% most frequently inputted obsessional words and for each “Group” of randomly-sampled users and their corresponding obsessions. The embeddings were trained on respective groups and reduced from 100 dimensions to 2 dimensions. The Elbow method’s optimal cluster for both groups occurs at point with greatest slope change (elbow): k = 3. The Silhouette method’s optimal cluster for both groups is determined by the highest coefficient score: k = 3. The Calinski-Harabasz method’s optimal cluster is determined by the highest score: k = 3. The Davies-Bouldin Index’ optimal cluster is determined by lower scores: k = 3.

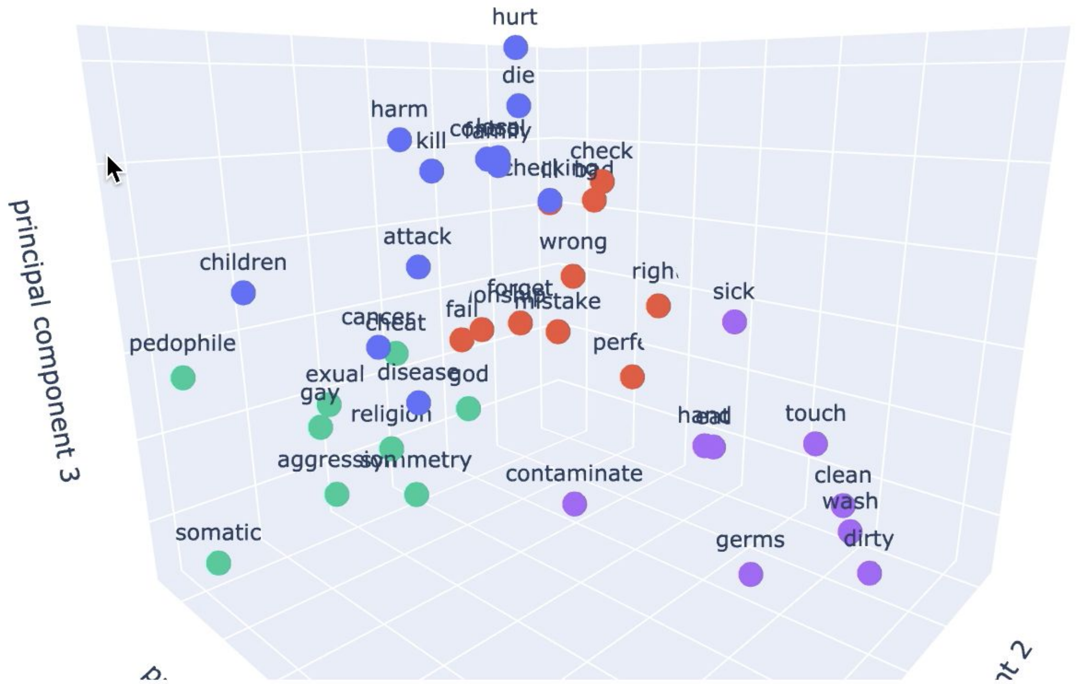

Fig. S26. 3D representation of canonical OCD-relevant words. To depict the semantic relationships of obsessional words in three dimensions, we performed data reduction of the 100 dimensional space to three dimensions using principal components analysis. As the top 7% of words that we used for the main analysis would result in an excessive number of overlapping dots and labels such that they would not be visible, we instead plotted the 35 obsessional words that frequently occur, clinically. For this, two psychiatrists (AL and JDF) each chose 35 OCD-relevant words out of the entire set of obsessional words that occur frequently based on their clinical experience. Words that were not in agreement were decided upon by a third clinician with OCD experience (MM) for a “tie-breaker,” to reach a final consensus.
*Please see the accompanying rotating video.
